# Geographic Patterns of Intra‐ and Interspecific Diversity of Riverine Fish Species in the Italian Northern Apennines and Ligurian Alps

**DOI:** 10.1002/ece3.73240

**Published:** 2026-04-03

**Authors:** Dominik Kirschner, Gabrielle Vance, Virginie Marques, Monika Goralczyk, Alice Valentini, Xiaowei Zhang, Sean D. Willett, Florian Altermatt, Loïc Pellissier

**Affiliations:** ^1^ Department of Environmental Systems Science, Institute of Terrestrial Ecosystems, Ecosystems and Landscape Evolution ETH Zürich Zürich Switzerland; ^2^ Department of Aquatic Ecology Eawag, Swiss Federal Institute of Aquatic Science and Technology Dübendorf Switzerland; ^3^ Department of Landscape Dynamics & Ecology Swiss Federal Research Institute WSL Birmensdorf Switzerland; ^4^ Department of Earth and Planetary Sciences, Geological Institute, Earth Surface Dynamics ETH Zürich Zürich Switzerland; ^5^ SPYGEN Le Bourget‐du‐Lac France; ^6^ State Key Laboratory of Pollution Control & Resource Reuse, School of the Environment Nanjing University Nanjing China; ^7^ Department of Evolutionary Biology and Environmental Studies University of Zurich Zürich Switzerland

**Keywords:** community ecology, environmental DNA, fish, landscape genetics, metabarcoding, phylogeography

## Abstract

Understanding the intricate dynamics of biodiversity within and across riverine ecosystems, influenced by geological history and environmental factors, is crucial for effective conservation and management strategies. Italy, particularly the Ligurian region, harbors diverse freshwater fish communities and populations shaped by unique geological and hydrological conditions. Here, we investigated the suitability of environmental DNA (eDNA) metabarcoding to identify inter‐ and intraspecific diversity patterns of riverine fish populations in drainage basins on both sides of the main drainage divide (MDD) between the Adriatic and Ligurian river basins in Northern Italy. We collected 96 aquatic eDNA samples across 48 riverine sites, amplified them using a cytochrome *b* primer pair, and denoised the sequences to retrieve amplicon sequence variants (ASVs). We calculated communities' phylogenetic distance with betaMPD based on genetic distances derived from the ASVs, combined them with conductance‐based landscape metrics, and applied generalized dissimilarity models to assess spatial genetic structure. Our results reveal genetic differentiation among populations of several fish species, with some displaying clustering patterns across the main drainage divide and isolation by distance patterns. Overall, taxon richness was higher on the Adriatic side (12.82 ± 3.57; 26 unique taxa) than on the Ligurian side of the MDD (8.35 ± 3.66 SD, 25 unique taxa), but the other way around for ASV richness across all species (Ligurian side: 51.94 ± 25.79, 308 unique ASV, Adriatic side: 68.00 ± 32.97, 274 unique ASV). Our findings highlight the effectiveness of eDNA metabarcoding in uncovering various facets of diversity, shedding light on hidden genetic diversity among ASVs, and revealing significant spatial genetic structuring in freshwater fish populations across multiple species.

## Graphical abstract

We investigated the suitability of environmental DNA (eDNA) metabarcoding to identify inter‐ and intraspecific diversity patterns of riverine fish populations across the Adriatic and Ligurian drainage basins in Northern Italy. Our results revealed genetic as well as community differentiations, with the Adriatic side displaying higher taxon richness while the Ligurian side harbored a higher number of unique amplicon sequence variants (ASVs). Overall, these findings highlight the effectiveness of eDNA metabarcoding in uncovering hidden genetic diversity and spatial structuring within freshwater fish communities.

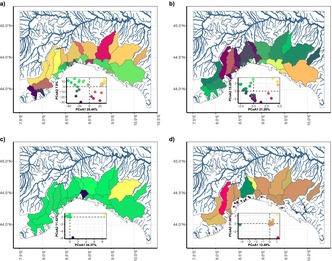

## 1 Introduction

Biodiversity is most commonly investigated at interspecific levels, usually focusing on taxonomic, functional, and phylogenetic diversity (Cadotte et al. 2011; Faith 1992; Jarzyna and Jetz 2016), but diversity extends from species to populations with intraspecific diversity. While intraspecific genetic diversity is typically less studied, it provides information on the evolutionary history of a species or community, can capture recent population dynamics and geographic differentiation not contained in taxonomic diversity, and can be critical in informing conservation and management strategies (Hoban et al. 2022, 2024). Intraspecific genetic diversity contains information on the state, history, and connectivity of populations and communities, which are directly linked to diversification and dispersal (Ochoa‐Ochoa et al. 2020; Webb et al. 2002). Mapping and measuring inter‐ and intraspecific diversity alongside each other could provide broader insight into the structure and history of populations and taxa. To date, combining inter‐ and intraspecific diversity in large‐scale studies has included labor‐intensive sampling efforts and invasive and costly capture methods; this process could be facilitated by the use of environmental DNA (eDNA).

The use of eDNA metabarcoding (Blackman et al. 2024; Pawlowski et al. 2020; Taberlet et al. 2012) has increased our ability to measure multiple facets of biodiversity (taxonomic, functional, and phylogenetic) across temporal (Djurhuus et al. 2020) and spatial scales (Civade et al. 2016; Kirschner et al. 2023; Marques et al. 2020) directly from environmental samples. eDNA can be retrieved from water, air, or soil, and is usually amplified with targeted or group‐specific primers and sequenced via high‐throughput sequencing to identify species and communities (Bruce et al. 2021; Creer et al. 2016; Taberlet et al. 2018), and is becoming an increasingly important technology toward reaching global biodiversity targets (Altermatt et al. 2025). While the reliable detection of community information is possible for some groups, such as fish or amphibians (Keck et al. 2022; Blackman et al. 2021; Hänfling et al. 2016; Brantschen and Altermatt 2024), detecting and validating intraspecific variation in eDNA metabarcoding datasets is known to be more challenging (Couton et al. 2023).

DNA metabarcoding is a valuable tool to examine intraspecific genetic diversity to gain phylogeographic insights and recover common haplotypes from aquatic eDNA (Macé et al. 2022; Sigsgaard et al. 2020; Tsuji et al. 2020, 2023) or, more commonly, from bulk samples collected by conventional methods (Antich et al. 2021; Shum and Palumbi 2021; Thomasdotter et al. 2023; Turon et al. 2020). The latter used a combination of generalist primers to detect genetic diversity from metabarcoding studies with some success, for instance, by reconstructing phylogeographic structure of marine benthos communities. Nonetheless, current aquatic eDNA metabarcoding studies on intraspecific diversity mainly rely on species‐specific primers, which require labor‐intensive primer design, limit the application to certain species, or require prior knowledge of existing haplotypes (Tsuji et al. 2023; Yatsuyanagi et al. 2024; Zanovello et al. 2023). Despite the potential of eDNA metabarcoding for detecting intraspecific variation, challenges such as false positives through PCR and sequencing errors, mistagging or tag jumping (Corse et al. 2017; Drake et al. 2022; Taberlet et al. 2018), limited genetic variation due to short mitochondrial markers (Miya et al. 2015; Valentini et al. 2016), or the inability to link the eDNA signal with individual organism information (Couton et al. 2023) must be considered. While the resolution of eDNA metabarcoding‐derived information at the intraspecific level falls short in comparison to modern‐day genomics using whole genome sequencing, the use of standard markers used in phylogeographic studies (e.g., cytochrome *b* and COI), along with novel bioinformatic tools to denoise sequences (Brandt et al. 2021; Callahan et al. 2016; Macé et al. 2022) holds promise. Used together with phylogenetic beta diversity metrics (Graham and Fine 2008; Webb et al. 2002), it holds the advantage of scaling up across entire communities, being non‐invasive, and may have enough power to assess landscape‐scale hypotheses (Shum and Palumbi 2021). As obligatory aquatic organisms, freshwater fish are bound to their surrounding matrix as habitat, are confined to dispersal along the waterways, and therefore exhibit specific hierarchical local and global diversity patterns (Muneepeerakul et al. 2008; Oberdorff 2022; Oberdorff et al. 1995, 2011; Tedesco et al. 2017). This is particularly true for primary riverine fish species unable to disperse through brackish or marine systems (Darlington 1948; Myers 1949). Rivers, as opposed to terrestrial or marine systems, are hierarchical dendritic networks, with strong biotic and abiotic gradients influencing the dispersal capabilities of organisms resulting in an island‐like structure of each river basin (Altermatt 2013; Oberdorff et al. 1995; Tonkin et al. 2018). This leads to diversity patterns within drainage networks, with riverine fish following upstream‐downstream gradients as well as gradients between basins, where geographically close (Euclidean distance) sites can be inhabited by different sets of species (Oberdorff et al. 1995, 2011). The connectivity within and among basins is evident in the high levels of genetic diversification within riverine species (Schmidt and Schaefer 2018) and can be linked to the geological and climatic history of the drainage network (Boschman et al. 2023; Dias et al. 2014).

Italy hosts a varied freshwater fish fauna shaped by complex colonization and diversification histories (Bianco 1995; Buj et al. 2017; Lorenzoni et al. 2021; Marchetto et al. 2010; Schmitt et al. 2021). While the overall fish fauna is well‐described, there is only limited data on fish distribution in small Ligurian basins and headwater streams within the Northern Apennines and Ligurian Alps (Sibilia et al. 2019). The short coastal basins are considered to lack primary fish fauna and the local communities were not established naturally (Bianco 1995, 2014). Recent studies challenge assumptions about the lack of native fish species in these areas, identifying primary cyprinid populations in Ligurian basins, likely spreading across the main drainage divide (MDD) (Lorenzoni et al. 2021; Marchetto et al. 2010; Zaccara et al. 2019). To assess the intra‐ and interspecific distributions of fish along the Ligurian coast, our eDNA sampling strategy targets the transition zone between the Ligurian Alps and the Northern Apennines, traversing the MDD separating the Ligurian and Adriatic Seas. This geographic feature, acting as a natural barrier for fish, offers a promising opportunity to uncover prevailing intra‐ and interspecific diversity patterns. To investigate this, we used mitochondrial cytochrome *b* gene‐based metabarcoding to study taxa distribution, the same cytochrome *b* fragment to investigate intraspecific variation on the amplicon sequence variant (ASV) level, and a phylogenetic diversity metric to identify spatial patterns. We predicted that (1) the beta diversity patterns differ between ASV diversity and genetic distance‐weighted intraspecific diversity; (2) our marker allows us to derive intraspecific genetic beta diversity for some fish species; (3) there are congruences in spatial patterns within and across species; and finally (4) that these intraspecific patterns show isolation by distance (IBD), indicating non‐random patterns within intraspecific eDNA data.

## 2 Materials and Methods

### 2.1 Sampling

Sampling took place in spring from 20.05.2021 to 30.05.2021 and 11.04.2022 to 20.04.2022. We selected sites to represent the Ligurian coast and paired them with sub‐basins of the Po River catchment, which ultimately drain into the Adriatic Sea (Figure S1). Environmental DNA was filtered using a peristaltic pump (Athena; Proactive Environmental Products LLC, Bradenton, Florida, USA) connected to a single‐use filtration capsule (VigiDNA 0.45 μm; SPYGEN, Le Bourget du Lac, France) by single‐use tubing. At each site, we took one sample of approximately 30 L of water (30 min sampling at 1 L/min) on the left and right bank of the river. Due to logistical difficulties in 2022, we altered the protocol and sampled 2 × 30 L on the same bank of the river rather than on both sides (Table S1). In total, we collected 96 samples of aquatic eDNA at 48 sites across 28 basins. When the filtration was completed, the filtration capsule was emptied of water, filled with 80 mL of CL1 conservation buffer (SPYGEN), and stored at room temperature until the DNA was extracted.

### 2.2 Laboratory Analysis

The eDNA extraction was performed following the procedure described in Pont et al. (2018). To generate longer fragments, we decided to use a primer pair amplifying ~420 bp of the cytochrome *b* gene (Hänfling et al. 2016, fwd: ‘AAAAACCACCGTTGTTATTCAACTA’, rev: ‘GCDCCTCARAATGAYATTTGTCCTCA’). The DNA amplifications were performed in a final volume of 25 μL of amplification mixture, using 3 μL of DNA extract as the template. The amplification mixture contained 1 U of AmpliTaq Gold DNA Polymerase (Applied Biosystems), 10 mM Tris–HCl, 50 mMKCl, 2.5 mM MgCl 2, 0.2 mM of each dNTP, 0.2 μM of each primer primer and 0.2 μg/μL bovine serum albumin (BSA, Roche Diagnostic). The primer sets were 5′‐labeled with an eight‐nucleotide tag unique to each sample (with at least three differences between any pair of tags), allowing the assignment of each sequence to the corresponding sample during sequence analysis. The tags for the forward and reverse primers were identical. The PCR mixture was denatured at 95°C for 10 min, followed by 50 cycles of 30 s at 95°C, 30 s at 50°C, 1 min at 72°C and the final elongation step at 72°C for 7 min. Twelve PCR replicates were run per filter. After amplification, the samples were quantified using capillary electrophoresis (QIAxcel; QiagenGmbH) and purified using the MinElute PCR purification kit (Qiagen GmbH). Before sequencing, purified DNA was quantified again using capillary electrophoresis. All PCR products were pooled in equal volumes to achieve a theoretical sequencing depth of 500,000 reads per sample. In total, four libraries were prepared by DNAGensee (Le Bourget du Lac, France) using the TruSeq PCR‐Free kit (Illumina) and sequenced separately with a MiSeq sequencer (2 × 250 bp, Illumina, San Diego, CA, USA) on the MiSeq Flow Cell Kits (v3; Illumina), following the manufacturer's instructions. Six negative extraction controls and two negative PCR controls (ultrapure water) were amplified (12 replicates each) and sequenced in parallel to the samples to monitor possible contamination.

### 2.3 Bioinformatics and Data Analysis

We initially demultiplexed and trimmed primer sequences from our raw sequencing files using cutadapt with default parameters and allowing no mismatches. We denoised each library separately using the DADA2 R package (Callahan et al. 2016). Before merging, we filtered sequences using the filterAndTrim function with a minimum length of 100, truncated reads at 220 bases from both ends, set the truncation threshold to 2 (trunQ), and the maximum expected error rate to 2 (maxEE). Additionally, we did not allow sequences to contain ambiguous bases (N). Error rates were learned using the learnError function with standard settings, followed by the actual denoising using the function dada with the standard settings. Following denoising, we merged sequences using the function mergePairs and filtered chimeras out using the removeBimeraDenovo function from DADA2. Lastly, we assigned the ASVs to taxa using the Decipher R package (Wright 2015) with the IDTAXA function, scanning for hits in all orientations of the DNA sequence and employing a confidence threshold of 60. To train the classifier, we used the Midori database (MIDORI2_UNIQ_NUC_GB253_cytb_RDP, Leray et al. 2022) accessed in February 2023. Our classifier underwent three training iterations to ensure robust performance, as recommended in the function's guidelines. Subsequently, the resulting raw ASV table underwent further filtering, where we retained only ASVs assigned to Actinopterygii, not flagged as pseudogenes, occurring in at least two samples, cleaned for contamination and having at least 10 reads overall. To address the detected low levels of contaminants in the extraction of negative controls, we subtracted reads per ASV based on their occurrence in blanks. For each ASV detected in a negative control, we subtracted the number of reads observed in that control from the read count of that ASV in all other samples. This subtraction was applied on a per‐ASV basis and not globally across the dataset. To account for the potential of pseudogenes or nuclear copies of mitochondrial genes, we aligned all ASV sequences to a *Salmo salar* cytochrome *b* sequence (NCBI: NC_001960.1), removed the first 15 bp of the 5′ end, and used the “translate” function of DECIPHER with the mitochondrial amino acid codes. We identified all ASVs sequences containing stop‐codons and removed them from further analyses (Tables S2 and S3). Finally, we excluded all marine fish families (Clupeidae, Mugilidae, Sparidae, Moronidae, Engraulidae, Scombridae, Xiphiidae, Carangidae, Istiophoridae) from the dataset. Further analyses were conducted at the basin level, necessitating the merging of multiple sites within the same (sub‐)basin for downstream analyses.

### 2.4 Landscape Metrics

To prepare the landscape metrics for general dissimilarity models (GDMs), we used Digital elevation models (DEMs) from the geodata R package (Hijmans et al. 2023) and the Hydro‐rivers raster from the HydroBASIN database (Lehner and Grill 2013) to calculate least‐cost‐paths (LCP) between basin centroids using the gdistance R package (van Etten et al. 2023). This creates transition rasters based on conductance to define the resistance of a landscape, where low values indicate low cost and high values result in high cost to move from a given cell to the next. We calculated three distance metrics: Euclidean distance (air distance between basin centroids), topographic‐like distance (following the river system), and topographic‐like distance weighted by elevation to account for river size. We defined conductance for topographic‐like distance (following the river system) in the following way: water bodies received a high conductance of 50,000; landmasses were marked with a lower conductance of 0.0001, representing an almost impenetrable barrier; and marine waters were given an intermediate conductance value of 150. We also calculated traversal costs based on topographic distance weighted by elevation: elevations below 100 m received a high conductance value of 1,000,000; those between 100 and 500 m, 100,000; 500 to 1500 m, 1000; between 1500 and 2000 m, 100; and elevations at or above 2000 m were set with a minimal conductance of 0.001. This method allowed us to conduct a nuanced analysis, integrating elevation‐based conductance and specifying distinct values for water and land, thereby facilitating comprehensive assessment of geographical connectivity and barriers within the study area. We then transformed the cost distances into geographical distances in kilometers.

### 2.5 Interspecific Analyses

To investigate if the Ligurian basins harbor different communities and higher species richness compared to the Adriatic basins, we transformed the ASV‐read matrix to a binary presence‐absence matrix. On the basis of the remaining freshwater taxa, we then calculated species richness and Jaccard's dissimilarity for the ASV composition as well as for the highest assigned taxon (which was only kept if it was at least at family level). We then used principal coordinate analysis (PCoA) to visualize the dissimilarities among communities and tested for differences between Adriatic and Ligurian basins using ANOSIM in the R package vegan (Oksanen et al. 2022) using the standard setting, 999 permutations, and the “Jaccard” distance.

### 2.6 Intraspecific Analyses

We investigated the potential of eDNA data to assess intraspecific diversity structure. We adopted a conservative approach to avoid overestimation of diversity due to errors arising from sequencing and PCRs, and thus applied common filtering thresholds (see bioinformatics section) and only kept ASVs with species level assignments. Analysis was done in R 4.3.2 (R Core Team 2024) using picante (function comdist, Kembel et al. 2010), pegas (Paradis et al. 2023), msa (function msa, Bodenhofer et al. 2015), and ape (function gendist, Paradis et al. 2023).

We aligned sequences for the available species using the msa function with ClustalO as the alignment algorithm. In the next step we used the gendist function to get pairwise genetic distances between aligned sequences. To combine this information with the occurrence data, we calculated the mean pairwise genetic dissimilarity between two sites (betaMPD, Webb et al. 2002) using the comdist function. We generated a PCoA ordination to visualize genetic distances between basins for each species using the cmdscale base R function. Then, we created maps by joining the ordinations with the basin information, visualizing the spatial distribution of genetic dissimilarity.

We created a haplotype network for the species *Telestes muticellus* , also incorporating available haplotype data from Zaccara et al. (2007). We aligned the sequences using the msa function of the package msa as before. We then visually inspected the alignment and trimmed the sequences to the overlapping region of the ASVs sequences and the haplotypes from Zaccara et al. 2007, resulting in a 302 bp fragment for the haplotype network analyses. We used the pegas package and its haplotype and haploNet (default settings using an infinite site model) functions to get haplotypes and frequencies (number of occurrences in the dataset), which we then visualized in a haplotype network.

### 2.7 Link Between ASV Identity and Genetic‐Weighted betaMPD

To test if the dissimilarities derived from ASV composition and the sequence‐weighted betaMPD held similar information, we used Mantel correlation analysis. We used the function Mantel in the package vegan (Oksanen et al. 2022) with the standard settings calculating the “pearson” correlation coefficient and 999 permutations.

### 2.8 Spatial Patterns of Inter‐ and Intraspecific Variations

To identify if the calculated intraspecific distances (genetic dissimilarities in the form of betaMPD) followed the expected spatial patterns, in this case isolation by distance (IBD), we applied GDMs (Rosauer et al. 2014) with the “gdm” package in R (Fitzpatrick et al. 2022) to all the species and distances. As input parameters, we used the betaMPD values calculated previously (which we scaled between 0 and 1 to follow one of the models' assumptions) and calculated one model per species and type of geographic distance created in LCP. These models were: Euclidean distance, LCP topography, and LCP along water weighted by elevation.

## 3 Results

### 3.1 Sequence Information

After denoising and taxonomic assignment, we detected a total of 13,757,653 raw reads from all the samples with a mean of 132,285 ± SD 149,341 reads per sample. We found a total of 2761 ASVs (mean read number 4983 ± SD 68,324), of which Actinopterygii dominated at 86.9%, followed by Mammalia at 10.3%, while other classes like Lepidosauria and Amphibia represented smaller proportions, each below 1%. Additionally, there were 52 instances where the class assignment was not available (NA), accounting for 1.88% of the total assigned ASVs.

### 3.2 Taxonomic Patterns

**FIGURE 1:**
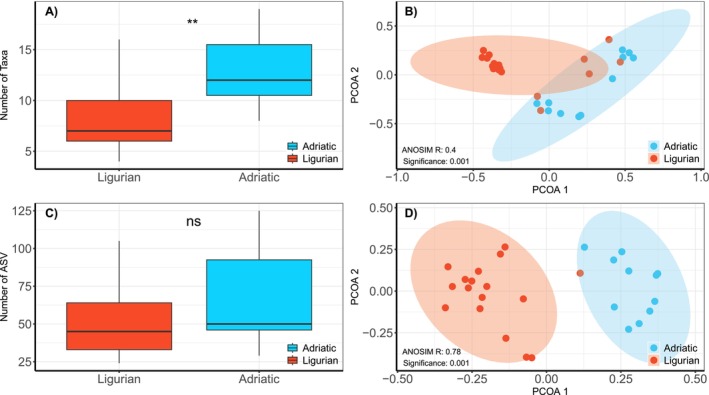
Comparison of taxa‐based data and ASV‐derived communities from the cytochrome b dataset. (a) shows the difference in taxon richness between both sides of the MDD. The ** indicate that there is a significant difference between both sides. (b) PCoA based on Jaccard's dissimilarity. (c) Difference in richness of ASVs assigned to fish on both sides of the MDD. (d) is the equivalent ordination based on Jaccard's dissimilarity. The ANOSIM results indicate that the community compositions are significantly different on both sides of the main drainage divide. Only freshwater fish or fish with freshwater life stages are included in this analysis.

After excluding non‐fish, all marine families, ASVs occurring in fewer than two samples, ASVs with fewer reads than in negative controls and represented by less than 10 reads, ASVs containing stop codons and subsequent pooling of all sites per river basin, we found 450 freshwater fish ASVs across 31 different taxa, of which 222 ASVs were detected in both years, and 228 only in one of the 2 years (2021: 201, 2022: 27). Within the river basins, the number of reads varied between 48,726 and 1,217,863 (mean = 390,222 ± SD 312,801). The number of ASVs detected per basin ranged between 24 and 125 ASVs and from 4 to 19 taxa assigned to at least family level (Table S4). In total, taxon richness was higher on the Adriatic side (26) compared to the Ligurian side of the MDD (25), while ASV richness showed the opposite patterns (Adriatic: 274, Ligurian: 308). On average, the Adriatic side of the MDD also showed significantly higher taxon richness than the Ligurian basins (Figure 1a, Wilcox *p* = 0.005). We found a similar, but in this case non‐significant, trend for ASV richness (Figure 1c, Wilcox *p* = 0.15). We tested whether the taxonomic as well as the ASV‐based community composition differed between the two sides of the MDD. The taxonomic community composition of both slopes differed significantly (Figure 1b, ANOSIM statistic R: 0.39, *p*‐value: 0.001). We observed a similar pattern for the ASV community composition (ANOSIM statistic R: 0.78, significance: 0.001) with even clearer separation between the Ligurian and Adriatic sides of the MDD (Figure 1d). Only a few taxa, such as *Telestes muticellus* , *Barbus plebejus*, and *Protochondrostoma genei*, showed widespread occurrence across the entire study area. Other taxa were more patchily distributed (e.g., *Barbus caninus* ) or occurred mainly on a single side of the MDD (e.g., *Phoxinus lumaireul* on the Adriatic side, Figure S4 and Figures S5–S21).

### 3.3 Intraspecific Results

**FIGURE 2:**
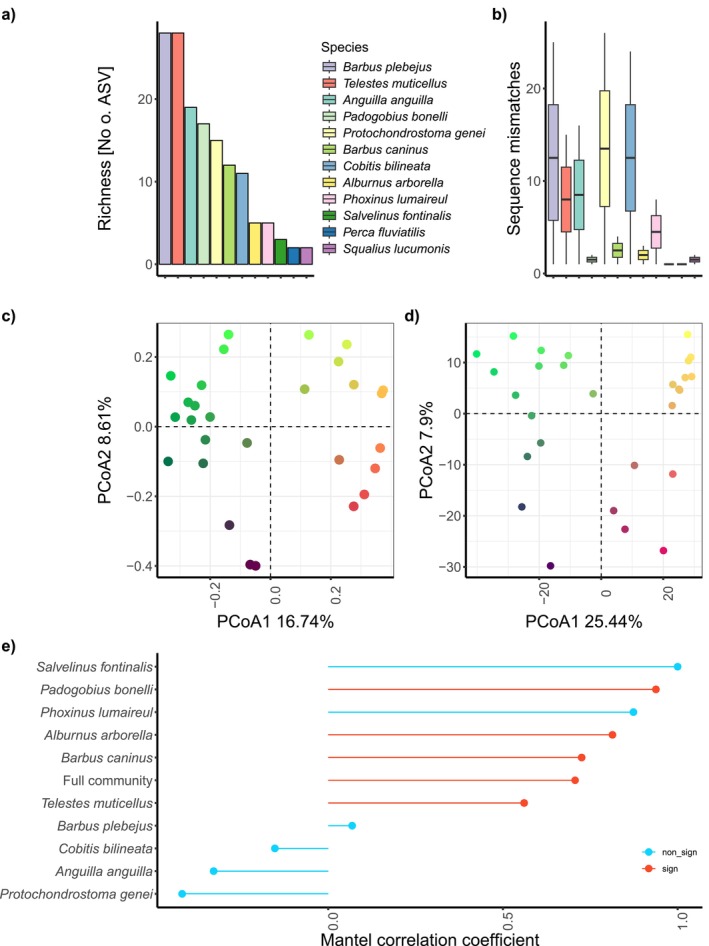
Overview of ASV richness, genetic distances, taxa‐ and betaMPD‐based PCoA, and correlation between Jaccard's dissimilarity matrix and betaMPD. (a) Number of ASVs per species; colors indicate the species. (b) Range of variation in genetic distance (as pairwise base pair mismatch) for the selected species. (c) PCoA of whole community Jaccard's dissimilarity based on ASVs. (d) PCoA based on betaMPD values for the whole community. (e) Correlation between ASV dissimilarities and betaMPD (genetic distance) per site, indicating variation among species.

Of all species detected, we focused on those with ASV occurrences in at least two basins, as this allowed pairwise comparisons needed for betaMPD. We ran intraspecific analyses on 10 species exhibiting coverage broad enough for the intraspecific analyses (*Barbus plebejus, Telestes muticellus, Anguilla anguilla, Protochondrostoma genei, Padogobius bonelli, Barbus caninus, Cobitis bilineata, Salvelinus fontinalis, Alburnus arborella, Phoxinus lumaireul*). In total, we found high variation in the number of ASVs, ranging from 91 ASVs for *Anguilla anguilla*, 65 for *Prochondrostoma genei*, 51 for *Telestes muticellus* down to 2 for *Salvelinus fontinalis (*Figure 2a). Within these ASVs, we found high variation of genetic distances (Figure 2b), with a mean genetic distance (measured as nucleotide difference) between sequences across all species of 4.20 (SD ±3.44). We observed the greatest genetic distances within the South European nase (*Protochondrostoma genei*, 1–26 bp) and the Italian barbel ( *Barbus plebejus* , 1–25 bp); and the lowest genetic distance in *Salvelinus fontinalis* and *Perca fluviatilis* .

Community‐wide betaMPD and ASV‐based Jaccard dissimilarities showed minor differences in the PCoA biplot (Figure 2c,d, Figure S2) and are correlated (Mantel correlation: 0.71, significance: 0.001, Figure 1e). This was true for most species, but the strength of the correlation coefficient varied between −0.42 and 1, indicating different patterns between species. For 6 of the 10 species, there was no significant correlation between betaMPD and ASV‐based dissimilarities (*Alburnus alborella, Anguilla anguilla, Phoxinus lumaireul, Cobitis bilineata, Salvelinus fontinalis*, and *Protochondrostoma genei*), which, with the exemption of *Anguilla anguilla* , are also the species with the lowest spread across the study area.

**FIGURE 3:**
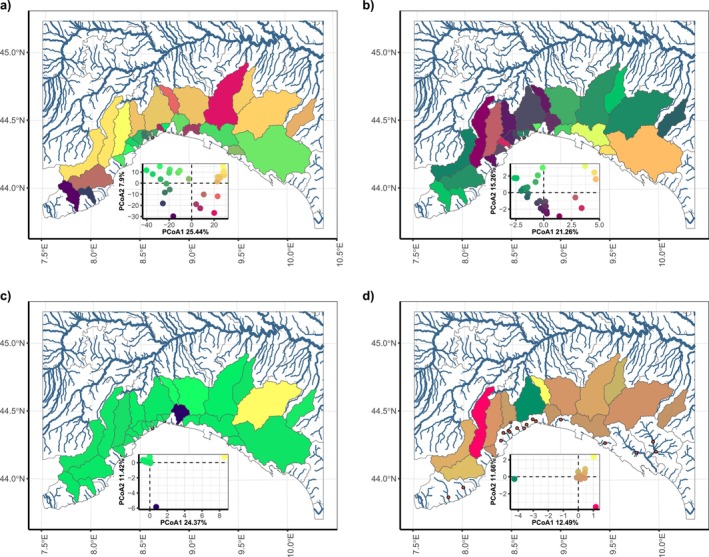
BetaMPD composition of different species with broad coverage across the MDD: (a) whole community MPD; (b) cyprinid *Telestes muticellus*, showing the strongest structuring; (c) cyprinid *Barbus plebejus* ; and finally (d) *Protochondrostoma genei*. Inlets show the corresponding PCoA for this species. Colors in the PCoA are the same as those in the map.

To identify how the betaMPD genetic dissimilarity patterns varied between species, we calculated PCoA based on betaMPD for every species with enough coverage and plotted them. The whole community betaMPD (Figure 3a) showed a separation between Ligurian and Adriatic basins. Overall, there was no congruent pattern that was valid across all species. For some species, like the Italian endemic *Telestes muticellus* , we found a marked structure and potentially three clusters of sites in the PCoA (Figure 3b). When mapped, there were two clusters in the Northern Apennines with the MDD as a barrier and a third cluster in the Ligurian Alps, which appeared to be more mixed across the MDD (Figure 3b). For *Barbus plebejus* and *Protochondrostoma genei*, two species also occurring throughout the whole study area, the structure was less visible and there were two basins that stood out distinctly (Figure 3c,d).

Incorporating known haplotypes from *Telesestes muticellus* collected by Zaccara et al. (2007), revealed a total of 38 haplotypes with some unique to this study, but also shared haplotypes (Figure S3 and Table S6). The haplotype frequencies range between 1 and 17 (Ht III). The analysis reveals genetic structuring between the Ligurian and Adriatic populations, with evidence of both shared and population‐specific haplotypes. Several haplotypes are exclusive to either the Ligurian or Adriatic basin populations, suggesting some degree of genetic differentiation. The presence of shared haplotypes indicates historical or ongoing gene flow between the two groups. A central haplotype (Ht III) appears highly connected, suggesting it potentially represents an ancestral or common variant from which others might have diversified. Overall, there is some congruence between known haplotype data and ASV‐derived data with central haplotypes such as Ht III or Ht XXVI being detected in this study, as well as in previous studies. Similarly to the genetic clusters of dissimilarity found in this study (betaMPD), we also see three main haplotype clusters in this network. Overall, the genetic relationships and mutational steps among haplotypes suggest moderate divergence with partial connectivity between Ligurian and Adriatic populations. These findings are broadly consistent with the haplotype patterns reported in earlier studies.

### 3.4 Spatial Structure

**FIGURE 4:**
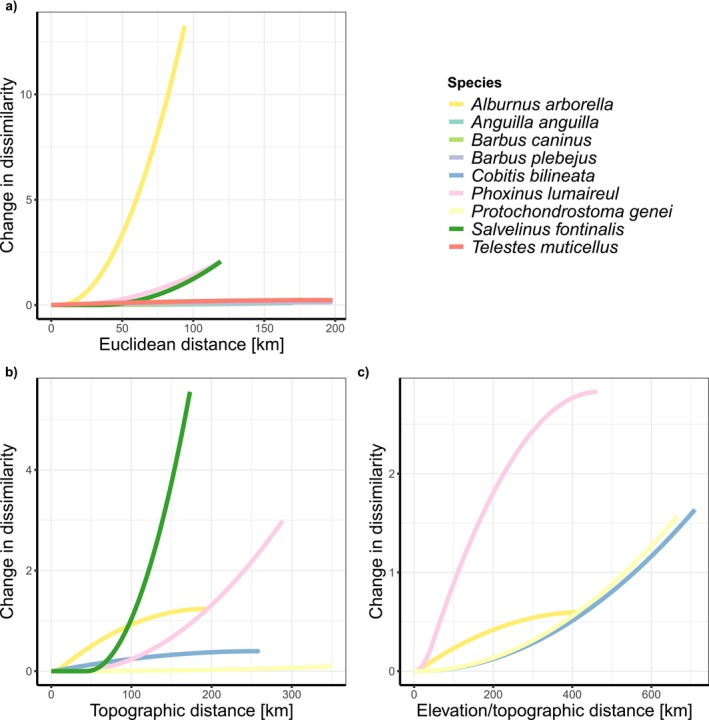
Results of the GDMs identifying significant links between (a) Euclidean distance, (b) topographic distance, and (c) elevation‐weighted topographic distance and dissimilarity gradients of taxa with species level assignment and sufficiently broad coverage.

To investigate spatial patterns and potential statistical support for non‐random spatial genetic structure in the data, we tested the isolation by distance hypothesis. Using GDMs, we found that Euclidean distance, topographic distance, and topographic distance corrected for elevation linked to changes in betaMPD for some, but not all species (Figure 4a–c). Species such as *Phoxinus lumaireul* show similar patterns of betaMPD change for topographic and elevation‐weighted topographic distances (Figure 4b,c). The explained variance for Euclidean distance varied between 0.06%–56.5%, for topographic distance between 0.1%–36.1%, and for elevation‐weighted distance between 1.0%–34.5% (Table S5). Overall, the slope and pattern of the I‐splines is quite variable, with extreme changes of dissimilarity for species like *Phoxinus lumaireul* in all distance metrics, and minimal changes along the Euclidean distance gradient for *Telestes muticellus* and *Barbus plebejus* (Figure 3a).

## 4 Discussion

Our study demonstrates that environmental DNA (eDNA) provides a versatile tool for comprehensive biodiversity detection of fluvial drainage basins of northern Italy covering both inter‐ and intraspecific diversity. Using cytochrome *b* as a marker, we identified taxonomic and intraspecific genetic diversity in the transition zone between the Northern Apennines and Ligurian Alps and revealed relatively high species richness, even in small basins. Notably, we observed a significant correlation in ASV composition patterns between classical identity‐based dissimilarity and genetic distance‐weighted betaMPD dissimilarity, suggesting overall congruence, but nuanced differences in inter‐ and intraspecific signals derived from eDNA. Particularly useful is the identification of spatial structure in eDNA‐derived intraspecific data, indicating non‐random patterns and isolation by distance.

### 4.1 Intraspecific Variation in the Study Area

We observed substantial genetic variation within ASVs assigned at the species level, particularly notable in species like *Barbus plebejus* and *Telestes muticellus* . For these species, we observed up to 26 bp differences on a ~420 bp long fragment. Still, these values are within known ranges of intraspecific variation in vertebrates such as fish (Li et al. 2018; Nicolas et al. 2012). When used to calculate betaMPD values, the overall patterns we find correlate closely with Jaccard's dissimilarity calculated on raw ASVs, but within species, more distinct patterns emerge. While the idea of isolated, island‐like river basins along the coast should lead to more distinct betaMPD patterns, we found similarity across the main drainage divide, especially west of Genoa. East of Genoa, and therefore within the Northern Apennines, we found a clearer separation into a Ligurian cluster and an Adriatic cluster. This was again clearest in *Telestes muticellus* , where we found roughly three clusters of similar sequences, potentially indicating different colonization histories, which are also supported by the overall shape and structure of the resulting haplotype network.

Our findings corroborate known phylogeographic patterns of fish species in Italy (Bianco 1995, 2014), where the interspecific results match the expected fish ichthyogeographic districts. The Ligurian basins east of Genoa are the most distinct in our data and fit the Tuscano‐Latinum ichthyogeographic district, while the communities north of the MDD, on the Adriatic side, fit the Padano‐Venetian district. For intraspecific variation, this congruence is harder to identify. Marchetto et al. (2010) found a cross‐divide population structure of *Telestes muticellus* between the Adriatic and Ligurian basins. However, this comparison needs some caution, because they used microsatellite data derived from individuals, while we investigated population‐wide genetic structure using cytochrome *b*. Still, the clusters we found in La Spezia, the more mixed populations east of Genoa in the Ligurian Alps, the structure of the haplotype network and the great sequence similarity across the MDD in the Ligurian Alps would partly fit their findings. Similarly, Zaccara et al. (2007) found phylogeographic clusters in the mitochondrial cytochrome b gene of this small cyprinid species. They could identify eastern Ligurian populations being separated from the Adriatic as well as the western Ligurian populations of *T. muticellus* . This aligns with our results, as these eastern Ligurian basins form a distinct intraspecific cluster. Even further, Zaccara et al. (2007) showed that certain haplotypes are shared across the MDD in the western Ligurian basins, which in our case too show more genetic similarity across the MDD. Indeed, when aligned to our dataset some of these haplotypes match the ASVs recorded in the present study and result in a clearly structured haplotype network. Despite the overall observed structure of the network, the observed reticulations in the network (e.g., around Ht XXV) may indicate the presence of unsampled “ghost” haplotypes or recurrent mutations (Bandelt et al. 2002). The comparison with Zaccara et al. (2007) confirms the biological validity of our sequences, while the detection of non‐overlapping haplotypes suggests that eDNA can capture diversity not currently represented in local reference sets. This highlights the potential for eDNA to complement traditional phylogeographic datasets, such as those by Buj et al. (2017), Dubut et al. (2012), and Ketmaier et al. (2004), to provide a more comprehensive resolution of fine‐scale biogeographic patterns.

For other species, like those within the genus *Barbus*, the structure is more complicated, as cryptic species in Italian barbels are common and their history is not yet fully resolved (Lorenzoni et al. 2021; Zaccara et al. 2019). Additionally, for species like *Phoxinus lumaireul* or *Alburnus alborella* we most likely underestimate true variation, as they are known to be quite variable on the cytochrome *b* gene (Caputo Barucchi et al. 2022; Reier et al. 2022) and our relatively low number of ASVs for this species could be the result of primer biases and problems of reference database coverage. It is still unclear how the Ligurian basins were colonized by primary species, as cyprinids are physiologically unable to disperse through marine waters (Darlington 1948; Myers 1949) and natural dispersal of obligatory aquatic species across drainage divides is difficult other than by river capture (Altermatt 2013; Tonkin et al. 2018). Besides the potential spread of some of these Cyprinid species during the Messinian Salinity Crisis, one explanation could be the region's geologic history, including glaciation in the Ligurian Alps (Rettig et al. 2024), which would have lowered sea level along the coast, and could have encouraged drainage reorganization; or the ongoing subduction and resultant asymmetric erosion across the orogenic wedge of the Northern Apennines (Erlanger et al. 2022; Willett et al. 2001) which encourages drainage reorganization including river capture across the main drainage divide (MDD). These geologic phenomena could contribute to taxonomic and genetic diversity patterns and population structure of certain fish species like *Telestes muticellus* (Marchetto et al. 2010; Zaccara et al. 2007). However, our results show that the intraspecific variation in this area is more complex than previously expected and probably needs further interdisciplinary efforts to understand the history of the Ligurian‐Adriatic MDD dynamics.

### 4.2 Taxon Richness in the Study Area

The higher richness observed in Adriatic basins aligns with expectations and is potentially influenced by basin size and geographic location. It is known that species richness can depend on basin size and differs between short coastal rivers and comparably sized tributaries of bigger catchments (Oberdorff et al. 1995, 2011). The Ligurian basins exhibit more similar taxonomic composition, likely due to the prevalence of some widespread freshwater genera like *Barbus* and *Telestes* across these regions. We see more variability on the Adriatic side of the MDD, most likely as a result of the overall larger species pool of the entire Po River catchment and marked distance among the different river branches (Bianco 2014; Sibilia et al. 2019). Opposite patterns can be found for the ASV richness, even though the difference between the Ligurian and Adriatic sides is small and potentially driven by the widespread occurrence and strong variability of *Anguilla anguilla* ASVs in the Ligurian basins. Generally, ASV‐based analyses are known to overestimate richness, but usually follow similar trends as taxonomic analyses, especially after quality filtering (Callahan et al. 2016, 2017). We find higher variability in ASV composition within Ligurian than Adriatic basins, especially along the small coastal basins. The Ligurian basins harbor a few species with exceptionally high ASV richness, including *Telestes muticellus, Barbus plebejus*, and *Anguilla anguilla* .

The ability to use metrics for comparing the evolutionary relationships among different biological communities to identify spatially nonrandom intraspecific variation from eDNA opens up new avenues to enhance large‐scale species inventories. Our study adds to the growing literature of eDNA and metabarcoding studies finding meaningful phylogeographic distributions (Antich et al. 2021; Turon et al. 2020) and recovering common haplotypes from eDNA (Adams et al. 2023). While most of the aquatic eDNA‐based studies so far designed primers specific to the species or genus to identify haplotypes (Andres et al. 2023; Sigsgaard et al. 2020; Tsuji et al. 2020, 2023), we used the same marker for intra and interspecific analyses. In line with existing literature, we showed that published primer sets targeting a sufficiently long fragment can harness aquatic eDNA for intraspecific patterns and variation (Shum and Palumbi 2021; Thomasdotter et al. 2023; Yatsuyanagi et al. 2024). We avoided potential issues with data analysis by utilizing denoised ASVs together with proper quality filtering and focusing on occurrence data instead of relying on read abundances, which are known to be influenced by various technical factors like PCR conditions or the applied primers (Rourke et al. 2022; Tsuji et al. 2019). Therefore, circumventing the use of read abundances avoids further bias in the data (Azarian et al. 2021). We suggest that because of the straightforward application of eDNA based methods in the field, adding taxonomic and intraspecific analyses to interdisciplinary studies (e.g., geology) could enable the broad‐scale identification of patterns across many species and direct efforts to better understand what shapes biodiversity.

### 4.3 Limitations of the Study and Analyses

Our results add to a growing body of literature showing the potential of adding eDNA into the toolbox for phylogeography or landscape genetics. Still, we need to acknowledge certain technical and analytical limitations. First, we found relatively high genetic distances, which may be indicative of some remaining PCR and sequencing errors which were not fully resolved despite our systematic quality filtering. Because we did not tag individual PCR replicates, we could not use these to filter our sequences further. In addition, recent literature shows that the introduction of mock samples as internal controls (e.g., positive controls with known DNA composition) would be of great benefit to help identify and mitigate these errors and should be considered already in the planning stage for future studies (Corse et al. 2017). The combination of negative and positive controls could enable a more data‐driven filtering step, informing on thresholds to be used to remove false positives while reducing false negatives (Drake et al. 2022) However, we detected 222 of 450 ASVs in both years. While this indicates that the core community is robust, the observed inter‐annual turnover is consistent with the stochastic nature of detecting low‐abundance eDNA signals (i.e., imperfect detection probability), rather than necessarily reflecting biological species turnover. Furthermore, while DADA2 is frequently and successfully used for taxonomic and intraspecific (Macé et al. 2022) analyses in metabarcoding studies, some further evaluation of its performance to identify “real” ASVs compared to alternative denoising and data curation tools should be considered given a recent critique (González et al. 2023).

Further, while arbitrary sequence thresholds (e.g., 10 reads) alone are probably not enough to remove all false positives, these thresholds are often enough to remove low‐frequency noise and other artifacts from sequencing (Drake et al. 2022) while retaining real sequence variants. In combination with data‐driven decision making to clean contaminations, this should be a good compromise in the absence of internal positive controls. Contamination and low‐frequency noise remain key challenges in metabarcoding workflows, particularly when interpreting ASV‐level diversity (Drake et al. 2022; Villsen et al. 2022; Goldberg et al. 2016). The removal of ASVs with internal stop codons and the subtraction of contaminant reads observed in negative controls helped refine our dataset. However, we acknowledge that fixed read thresholds, even when conservative, may inadvertently exclude genuine low‐abundance signals, especially in rare taxa. Conversely, global thresholds based solely on negative controls risk discarding valid ASV occurrences unrelated to contamination. In the absence of positive controls, our strategy reflects a compromise aimed at retaining biological signals while reducing artifacts. Future studies would benefit from integrating standardized mock communities to better calibrate such thresholds and improve comparability across studies (Corse et al. 2017; De Barba et al. 2014; Drake et al. 2022).

Second, most studies working on intraspecific eDNA target certain haplotypes or have previous knowledge about expected intraspecific diversity. We had no access to living individuals or tissue samples, but given the congruence of our results with patterns described in previous studies for small species of interest like *Telestes muticellus*, and the overall matches in the haplotype network between the known haplotypes and the recovered ASVs, we would argue that the ASVs we detect allow us to draw similar conclusions to those of classic haplotype‐based studies.

Third, anthropogenic influences probably shape vast parts of the Italian fish fauna (Lanzoni et al. 2018), and we could not fully account for them. However, some species like *Telestes muticellus* , a primary freshwater fish, can be seen as small enough with no value for fisheries and therefore no planned anthropogenic translocation (Bianco 1995, 2014). Still, there is evidence of anthropogenic translocation by anglers for the similar‐sized species complex of *Phoxinus*. These small species can be used as bait by anglers and could therefore be translocated by accident, leaving some uncertainty as to whether we see natural distributions in the whole study area (De Santis et al. 2021).

Fourth, we were not able to assign some of the taxa to species level. This is quite common in eDNA studies, even when reference databases are complete (Blackman et al. 2023; Keck et al. 2023). In the case of this study, we used the curated reference database MIDORI2 (Leray et al. 2022), which is maintained and updated regularly, but is potentially incomplete for some species (or haplotypic variation) in our study region. The lack of species level assignments for some taxa makes it challenging to analyze intraspecific variation across the whole dataset. For instance, the genus *Squalius* and *Barbus* are quite common in our dataset, and similar to many European Leuciscids and *Cyprinids*, they exhibit complex intraspecific structure (Rossi et al. 2021). While our species ID tool was able to assign some of the ASVs to species (e.g., *Squalius lucumonis*), we have no means to assign the other taxa reliably without introducing additional assumptions (e.g., biogeography) or and/or analyses (e.g., phylogenetic analysis; Corse et al. 2017). A similar lack of taxonomic resolution is likely due to haplotype sharing between closely related species, resulting from a recent and/or reticulate evolutionary history. In our case, this concerns *Barbus plebejus* and *B. tyberinus* (Rossi et al. 2021), as well as Alburnus arborella and *A. albidus* (Ketmaier et al. 2009). Such patterns likely led to an underestimation of both the occurrence and the genetic diversity of *B. plebejus* and A. arborella in our dataset.

One solution to this could be the addition of either biogeographical information on the available taxa, which would allow the decision for one species in a genus if it is the only one to be expected. For instance, we did not detect common species like *Cyprinus carpio* , *Barbus barbus, Oncorhynchus mykiss*, or *Salmo trutta* at the species level. We only detected those on a genus level, despite only one species per genus to be expected in the area. This is sometimes used in studies, but is not feasible when there is a lack of taxonomic expertise or phylogeographical knowledge, and hardly scalable in highly diverse areas (Blackman et al. 2021; Cilleros et al. 2019). Further, a second assignment step using complementary algorithms could be helpful (Corse et al. 2017), as all of the available methods have different weaknesses and strengths and could potentially be used complementarily (Hleap et al. 2021). However, this would add additional difficulties regarding computational availability, adding additional biases regarding the used algorithms' similarity thresholds, difficulties with ambiguous assignments, and increases the complexity of the workflow. We acknowledge that these systematic false negatives likely result in conservative estimates of species richness and may obscure fine‐scale connectivity patterns for these specific taxa. In our case, one of the benefits of the used method is the potential to train classifiers in advance, and then reuse them multiple times, making the assignment step fast and easily executable, even by non‐experts (Hleap et al. 2021; Murali et al. 2018). Therefore, we argue that this approach, while not showing the highest precision (in terms of assignment at the species level), makes eDNA metabarcoding valuable to get first insights into a region's intra‐ and interspecific diversity, and is scalable, reproducible, and valuable across disciplinary borders. The still remaining issues with precision and assignment quality in this step could be solved by stricter training of the classifier or better curated reference databases, including knowledge on existing haplotype variation, potentially addressing remaining uncertainties regarding precision.

Finally, our GDMs revealed low explained variance, suggesting that factors beyond isolation by distance may play significant roles in shaping intraspecific variation within the studied region. For instance, Euclidean distance does not take into account the topology or geometry of the river network between sites, which is of fundamental importance for fish species limited to the river channel (Altermatt 2013; Tonkin et al. 2018). By building least cost paths simulating topographic distances (with and without weight for elevation), we tried to account for this, as the river network is the most realistic corridor for gene flow. In our case, we even allowed for connections across the divide, mainly in low‐elevation areas with short distances, to potentially account for some level of (historical) connectivity across the MDD. Certain species appear to follow distance gradients, particularly cyprinids and leuciscids, despite their known inability to disperse through marine waters, but not all of the species exhibit similar patterns. This suggests a more complex interplay of factors influencing genetic structure within these Italian riverine systems' populations.

## 5 Conclusions and Outlook

In summary, our findings highlight the presence of significant intra‐ and inter‐specific information within eDNA metabarcoding data, particularly in the diverse ecosystem of the Ligurian coast. To improve the quality of the eDNA sequencing data for future intraspecific analyses, the addition of mock samples for contamination and more stringent error control (González et al. 2023), as well as combining multiple or more variable markers (Corse et al. 2019), could prove helpful. In addition to more variable markers, targeting specific genera of interest rather than whole groups (Yatsuyanagi et al. 2024) could improve the specificity of eDNA for genetic diversity studies. Furthermore, developments in the use of longer read sequencing for eDNA (Egeter et al. 2022; Tedersoo et al. 2021) could open new avenues to further develop the eDNA toolbox for phylogenetics and questions of intraspecific diversity. Finally, while first studies already demonstrated the use of eDNA metabarcoding derived occurrence data in population genetics (Shum and Palumbi 2021), establishing connections between genetic markers and read abundance data would allow for the application of abundance‐based population genetic metrics, enhancing our understanding of ecological processes at play on a large scale. Incorporating these advancements into future research promises to deepen our understanding of riverine biodiversity and would allow us to answer multidisciplinary questions between geology, ecology, and phylogeography, shedding light on the historical and recent connectivity of rivers and its geological drivers.

## Author Contributions

**Dominik Kirschner:** conceptualization (equal), data curation (lead), formal analysis (lead), investigation (lead), visualization (lead), writing – original draft (lead), writing – review and editing (equal). **Gabrielle Vance:** data curation (supporting), formal analysis (supporting), investigation (supporting), writing – original draft (equal), writing – review and editing (equal). **Virginie Marques:** formal analysis (supporting), writing – review and editing (equal). **Monika Goralczyk:** investigation (supporting), writing – review and editing (equal). **Alice Valentini:** investigation (supporting), writing – review and editing (equal). **Xiaowei Zhang:** supervision (supporting), writing – review and editing (equal). **Sean D. Willett:** conceptualization (equal), funding acquisition (equal), supervision (supporting), writing – review and editing (equal). **Florian Altermatt:** conceptualization (equal), resources (equal), supervision (supporting), writing – review and editing (equal). **Loïc Pellissier:** conceptualization (equal), funding acquisition (equal), resources (equal), supervision (lead), writing – review and editing (equal).

## Funding

This work was supported by Schweizerischer Nationalfonds zur Förderung der Wissenschaftlichen Forschung (100744200).

## Conflicts of Interest

A.V. is a research scientist in a private company specializing in the use of eDNA for biodiversity monitoring. All other authors declare no conflicts of interest.

## Supporting information


**Figure S1:** Overview of the Ligurian coast. Sampling sites are color‐coded according to their position on either side of the main drainage divide. Black outlines are the sampled drainage basins.

**Figure S2:** Comparison of community composition along the Ligurian‐Adriatic MDD. (A) PCoA depicting Jaccard's pairwise dissimilarity. (B) Mapped out PCoA values, colors correspond to values in (A). (C) PCoA based on betaMPD with (D) mapped out values from (C) with corresponding colors.

**Figure S3:**: Haplotype network for Telestes muticellus. The color of the bubble corresponds to the occurrence in either the Ligurian (red, green) or Adriatic (blue, purple) basins of the study area or the occurrence in Zaccara et al. 2007. The pie chart indicates the frequency of occurrence of these haplotypes on either side of the MDD. Dashed lines indicate potential alternative configurations.

**Figure S4‐S13:** Geographic distribution of different species based on ASV (Amplicon Sequence Variant) detections. The figure presents spatial maps displaying the occurrence of all ASVs across different locations in Northern Italy. The plotted regions include coastal areas and the Po River basin, distinguished by different colors. Longitude (°E) and latitude (°N) coordinates are provided to indicate the spatial extent of the study area. The underlying base map includes hydrological features such as rivers. The species shown did undergo filters, but not all of them were included in intraspecific diversity analyses.

**Table S1:** Overview of sampling metadata and sampling sites.

**Table S2:** Raw assigned ASV Table. Assignment was done using Decipher trained on the MIDORI2 database. Numbers are read numbers for this ASV within this sample.

**Table S3:** Raw assigned ASV Table after correcting for contamination by removing read numbers found in negative controls from ASVs, as well as removal of NUMTs. Assignment was done using Decipher trained on the Midori2 database. Numbers are read numbers for this ASV within this sample.

**Table S4:** Species and ASV richness along the Adriatic‐Ligurian MDD. The corrected ASVs are corrected for the number of sites within the basin.

**Table S5:** Summary of GDM outputs for different models.

**Table S6:** List of Haplotypes, corresponding ASV_IDs, slope of occurrence of the ASV and frequency per slope.

## Data Availability

Pending acceptance of this manuscript, the raw sequencing data, metadata, and bioinformatic code associated with this study will be stored and made available to the public through Zenodo (DOI: 10.5281/zenodo.18183624). For the purpose of peer review, the dataset can be accessed anonymously via the following link: Zenodo Reviewer Link. We are committed to ensuring the transparency and accessibility of our data to facilitate further research and collaboration.
