# Supplementary figures and images for: Geographic Patterns of Intra‐ and Interspecific Diversity of Riverine Fish Species in the Italian Northern Apennines and Ligurian Alps
Source: Ecol Evol. 2026 Apr 3;16(4):e73240. doi: 10.1002/ece3.73240 (PMC13052162; doi:10.1002/ece3.73240)

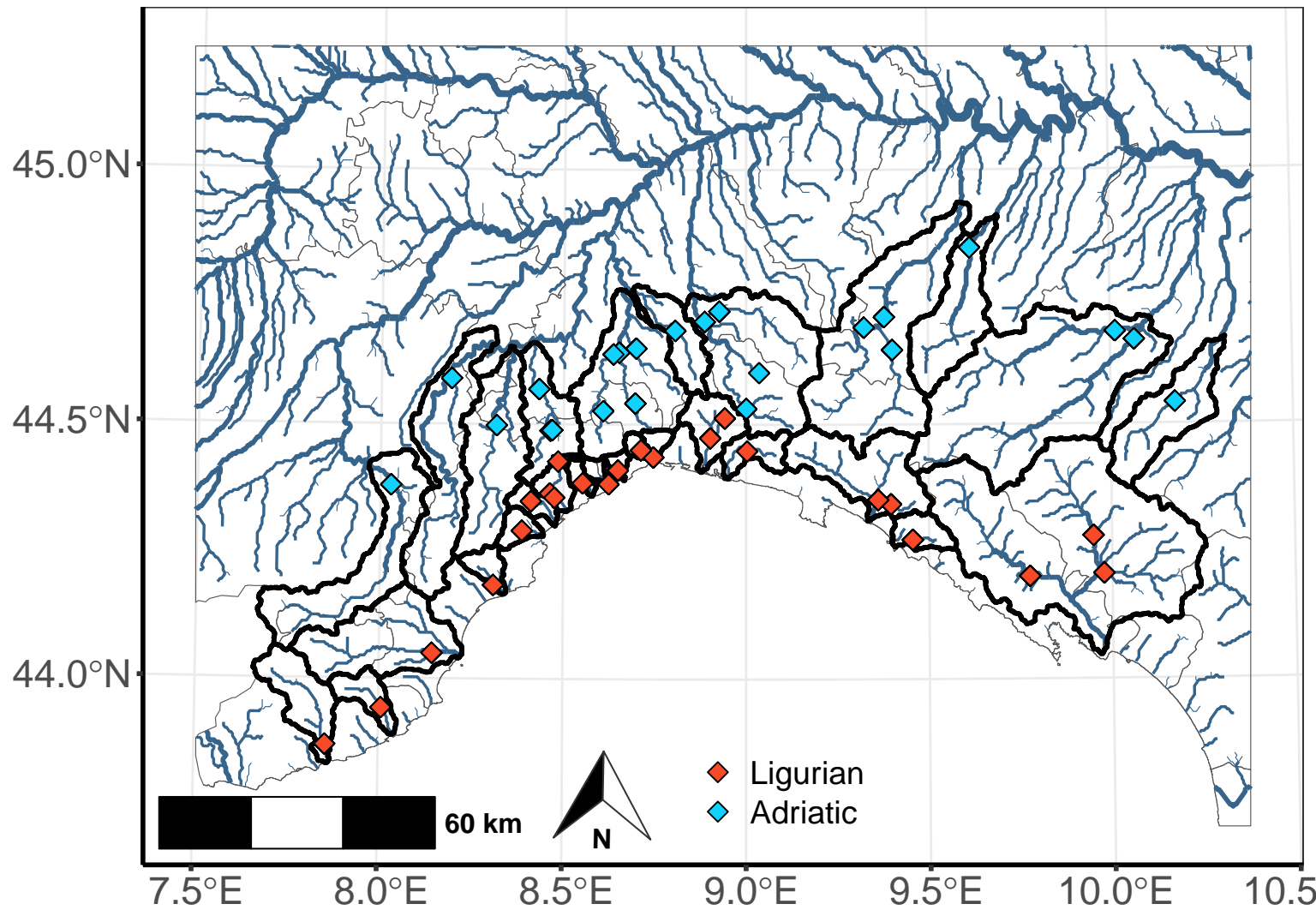

Supplement: Supplementary file 1 — Figure S1: Overview of the Ligurian coast. Sampling sites are color‐coded according to their position on either side of the main drainage divide. Black outlines are the sampled drainage basins. [file ECE3-16-e73240-s007.pdf]

**A) Jaccards dissimilarity – ASV**

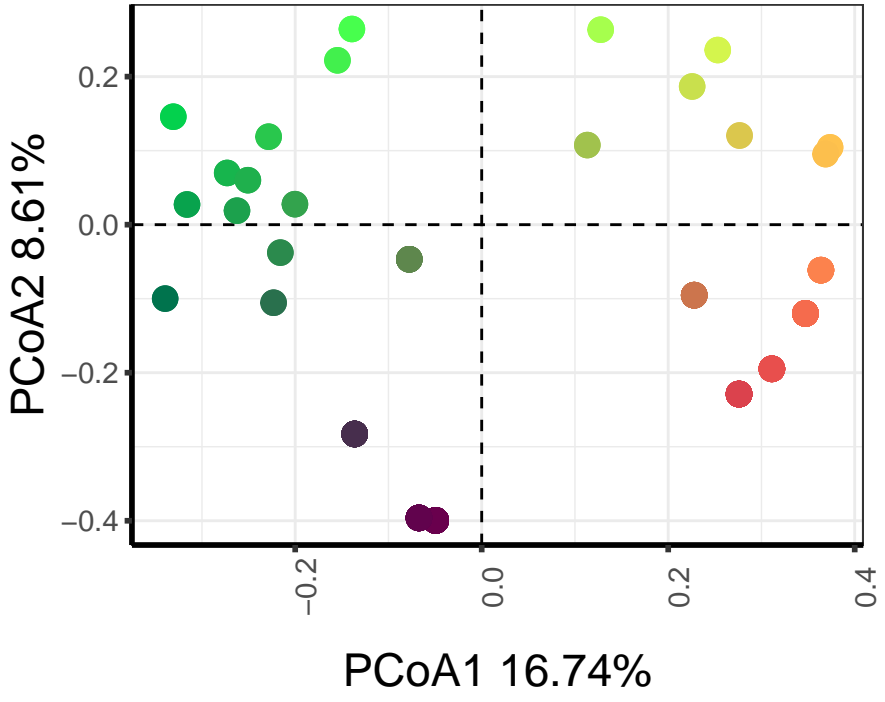

**B)**

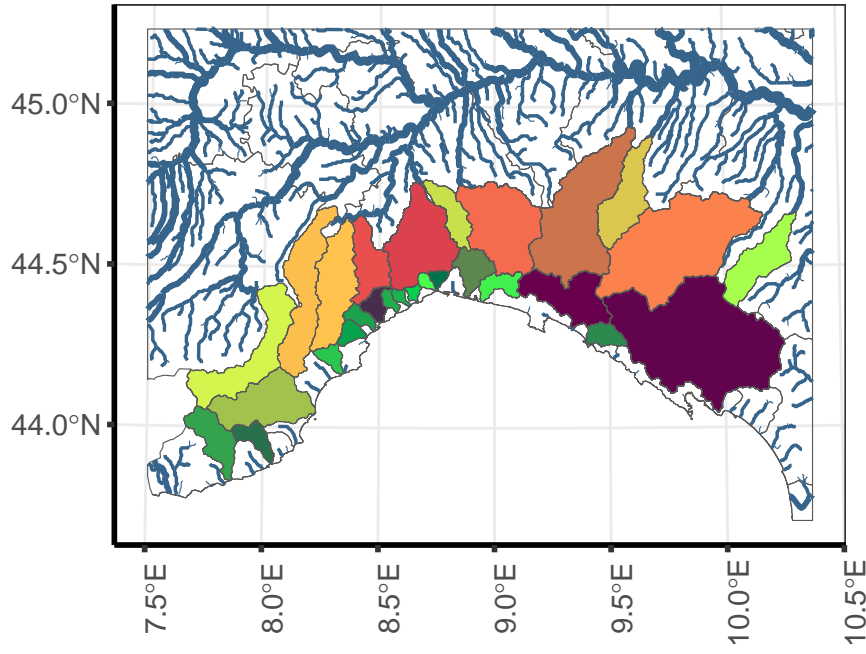

**C) Beta-MPD dissimilarity**

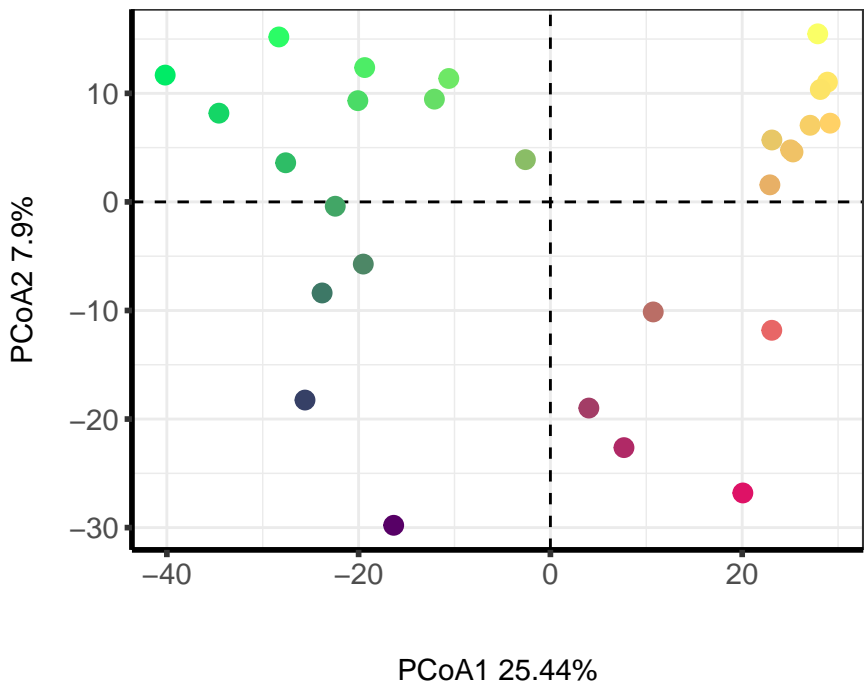

**D)**

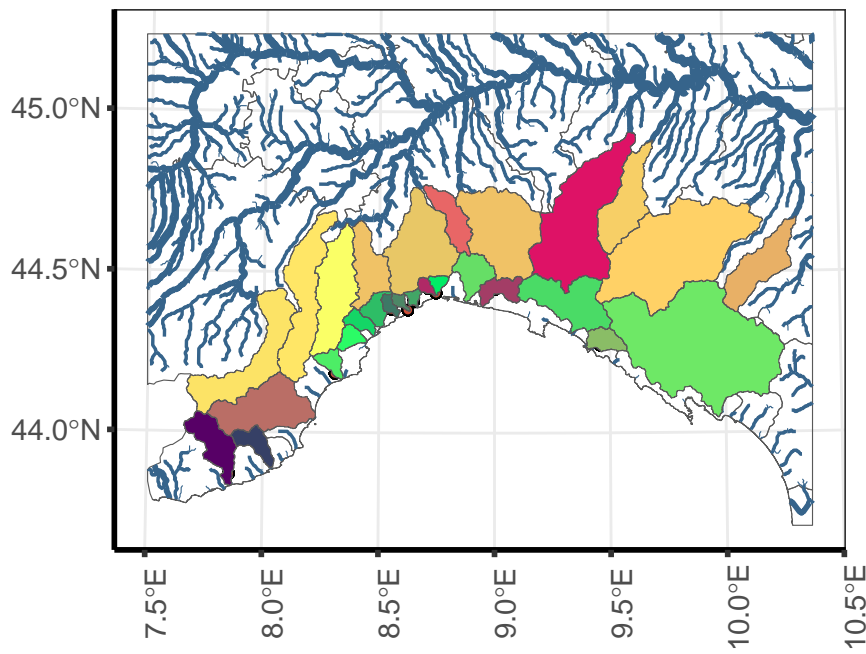

Supplement: Supplementary file 2 — Figure S2: Comparison of community composition along the Ligurian‐Adriatic MDD. (A) PCoA depicting Jaccard's pairwise dissimilarity. (B) Mapped out PCoA values, colors correspond to values in (A). (C) PCoA based on betaMPD with (D) mapped out values from (C) with corresponding colors. [file ECE3-16-e73240-s005.pdf]

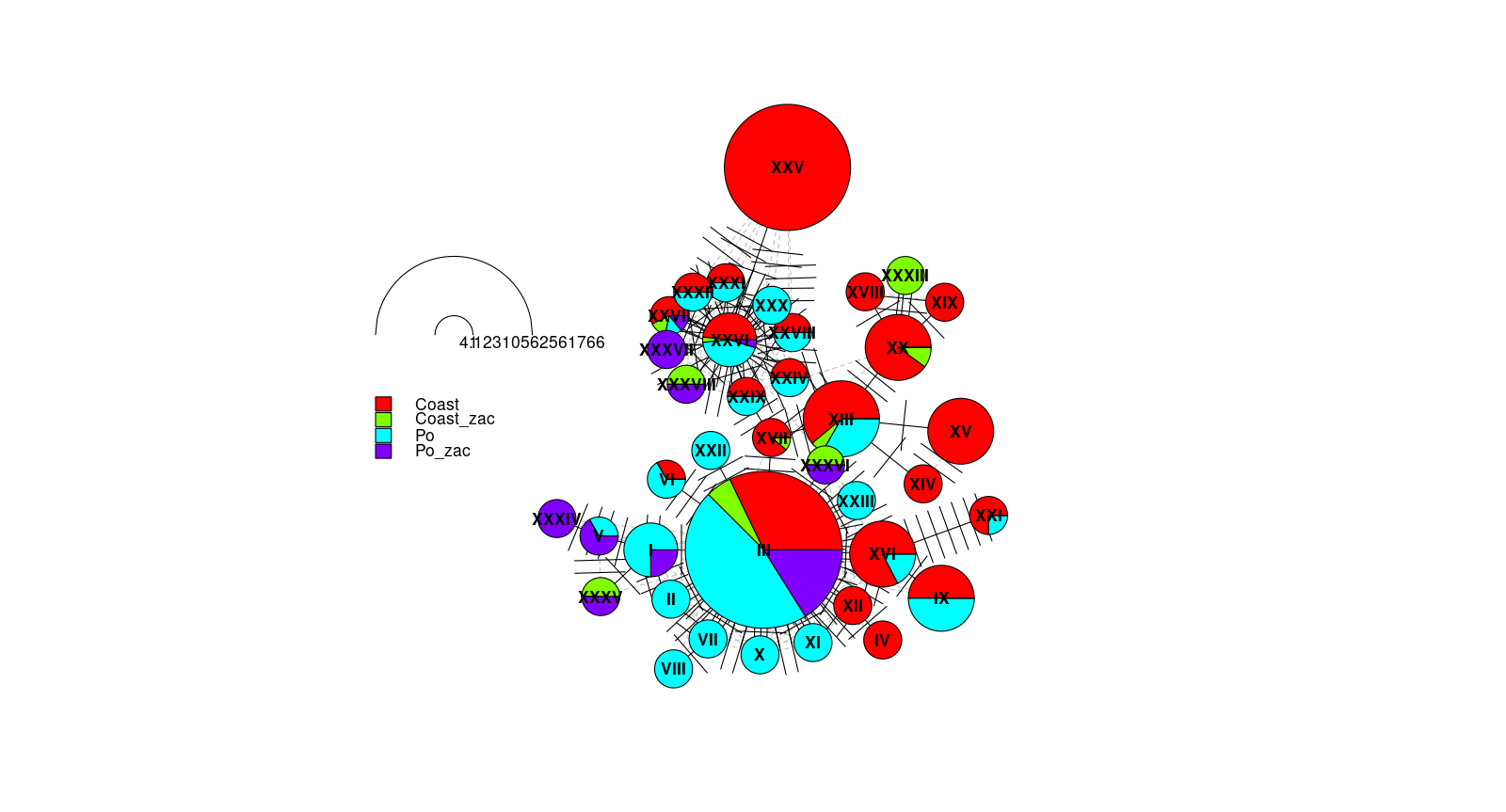

Supplement: Supplementary file 3 — Figure S3:: Haplotype network for Telestes muticellus. The color of the bubble corresponds to the occurrence in either the Ligurian (red, green) or Adriatic (blue, purple) basins of the study area or the occurrence in Zaccara et al. 2007. The pie chart indicates the frequency of occurrence of these haplotypes on either side of the MDD. Dashed lines indicate potential alternative configurations. [file ECE3-16-e73240-s009.png]
