# Supplementary material for: Geographic Patterns of Intra‐ and Interspecific Diversity of Riverine Fish Species in the Italian Northern Apennines and Ligurian Alps
Source: Ecol Evol. 2026 Apr 3;16(4):e73240. doi: 10.1002/ece3.73240 (PMC13052162; doi:10.1002/ece3.73240)
Supplement: Supplementary file 4 — Figure S4‐S13: Geographic distribution of different species based on ASV (Amplicon Sequence Variant) detections. The figure presents spatial maps displaying the occurrence of all ASVs across different locations in Northern Italy. The plotted regions include coastal areas and the Po River basin, distinguished by different colors. Longitude (°E) and latitude (°N) coordinates are provided to indicate the spatial extent of the study area. The underlying base map includes hydrological features such as rivers. The species shown did undergo filters, but not all of them were included in intraspecific diversity analyses. [file ECE3-16-e73240-s004.pdf]

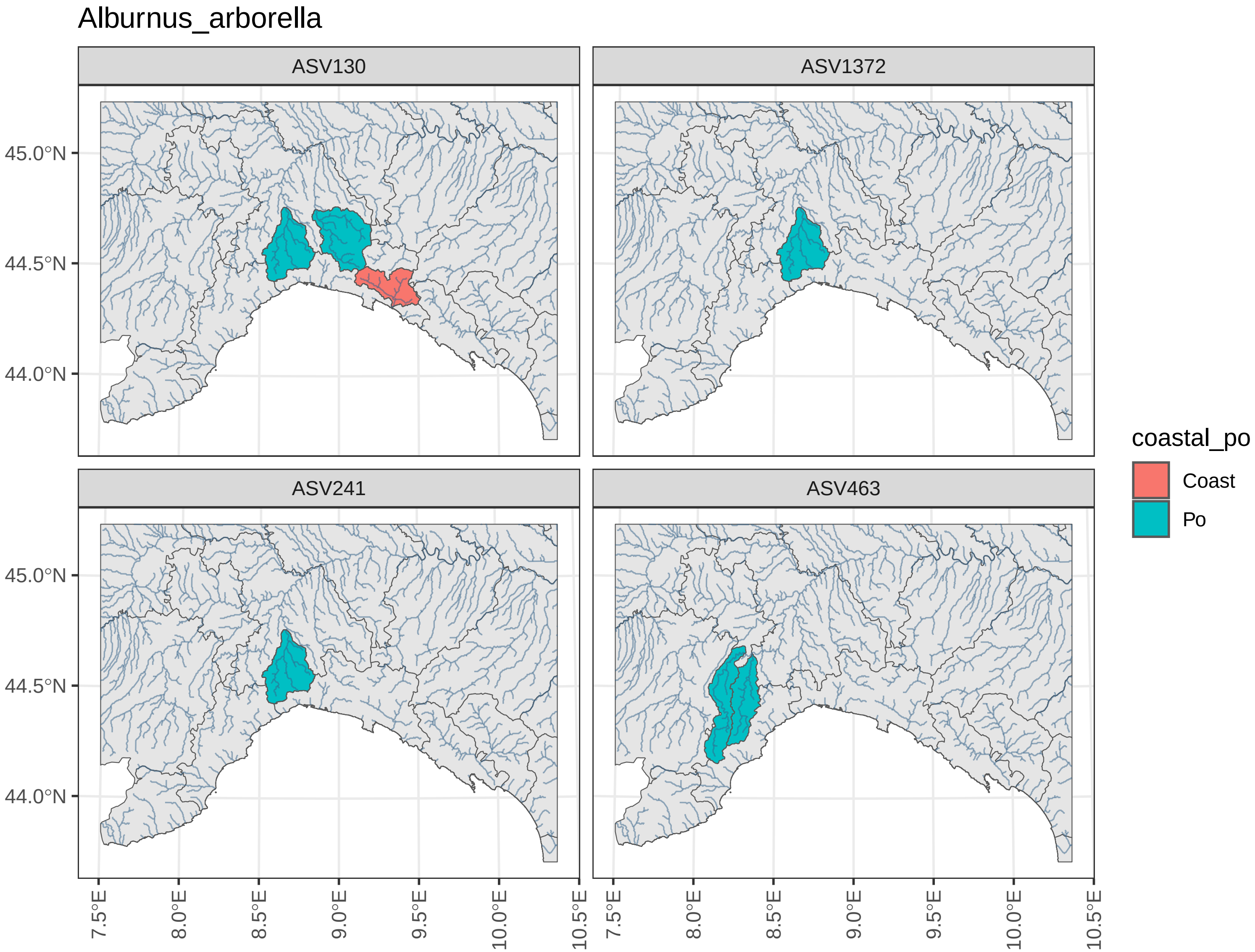
Alburnus_arborella
ASV130
ASV1372
ASV241
ASV463
45.0°N
44.5°N
44.0°N
7.5°E
8.0°E
8.5°E
9.0°E
9.5°E
10.0°E
10.5°E
coastal_po
Coast
Po

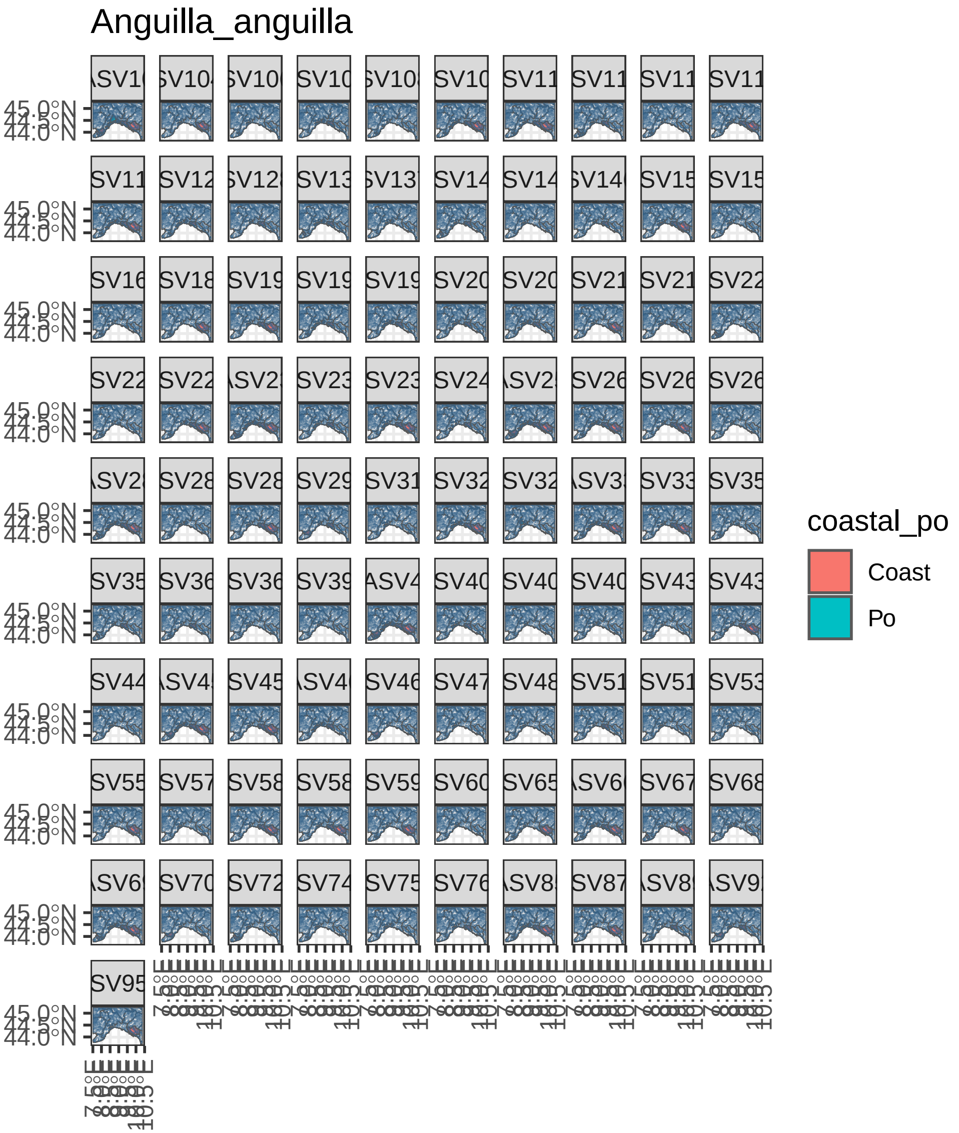
Anguilla_anguilla
coastal_po
Coast
Po
45.0°N
44.0°N

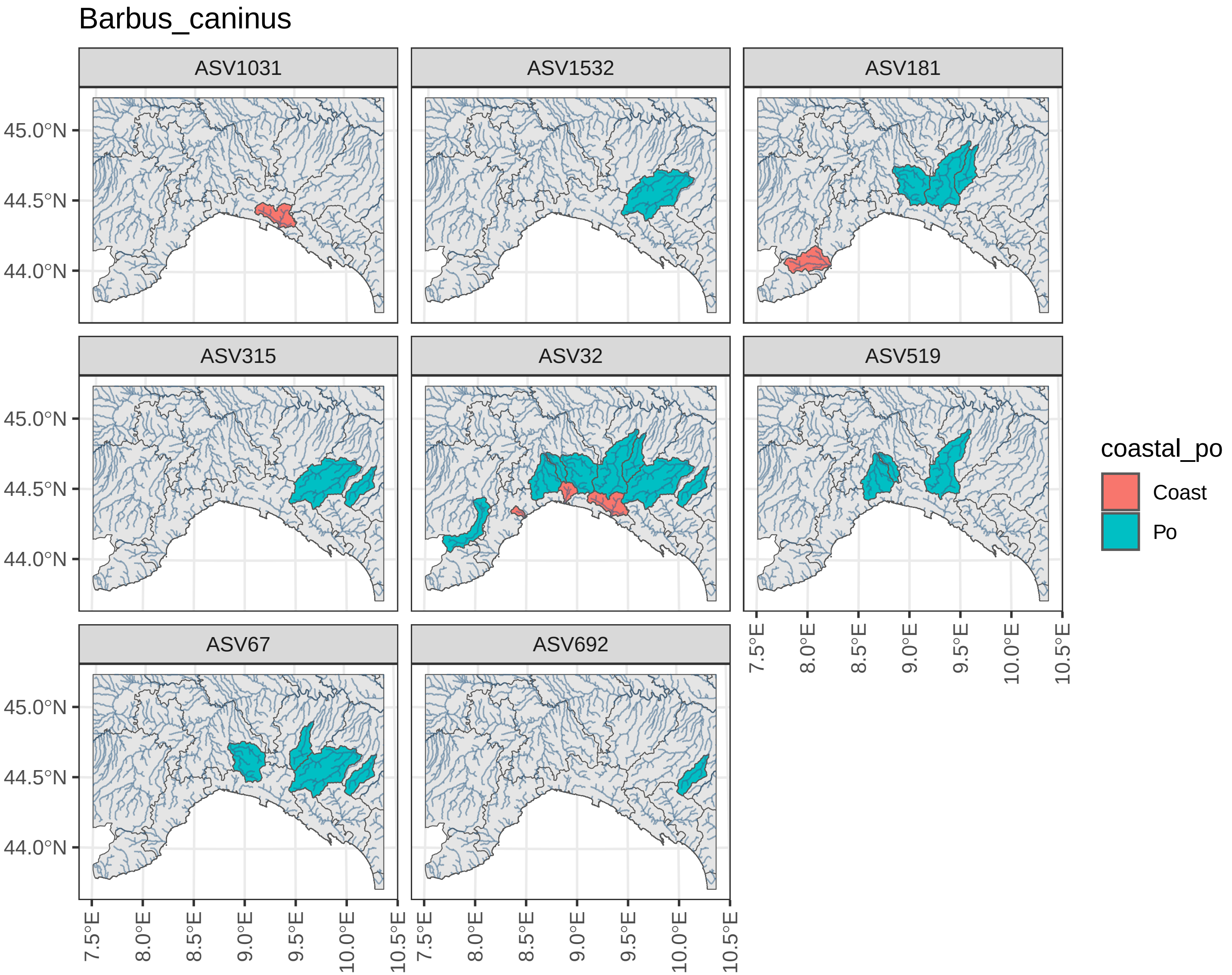

Barbus_caninus
ASV1031
ASV1532
ASV181
ASV315
ASV32
ASV519
ASV67
ASV692
45.0°N
44.5°N
44.0°N
7.5°E
8.0°E
8.5°E
9.0°E
9.5°E
10.0°E
10.5°E
coastal_po
Coast
Po

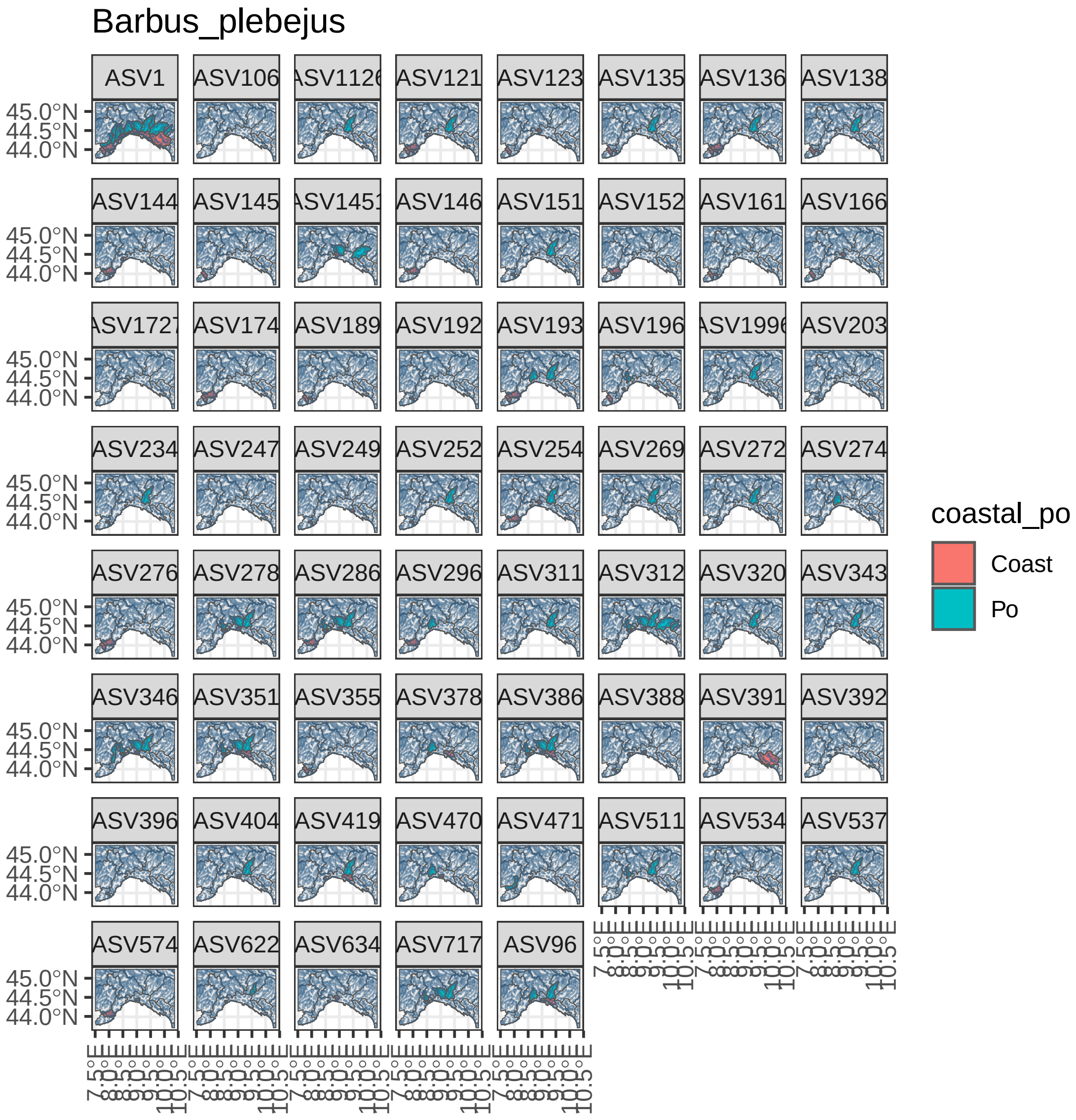

Barbus_plebejus
ASV1
ASV106
ASV121
ASV123
ASV135
ASV136
ASV138
ASV144
ASV145
ASV146
ASV151
ASV152
ASV161
ASV166
ASV174
ASV189
ASV192
ASV193
ASV196
ASV203
ASV234
ASV247
ASV249
ASV252
ASV254
ASV269
ASV272
ASV274
ASV276
ASV278
ASV286
ASV296
ASV311
ASV312
ASV320
ASV343
ASV346
ASV351
ASV355
ASV378
ASV386
ASV388
ASV391
ASV392
ASV396
ASV404
ASV419
ASV470
ASV471
ASV511
ASV534
ASV537
ASV574
ASV622
ASV634
ASV717
ASV96
45.0°N
44.5°N
44.0°N
coastal_po
Coast
Po

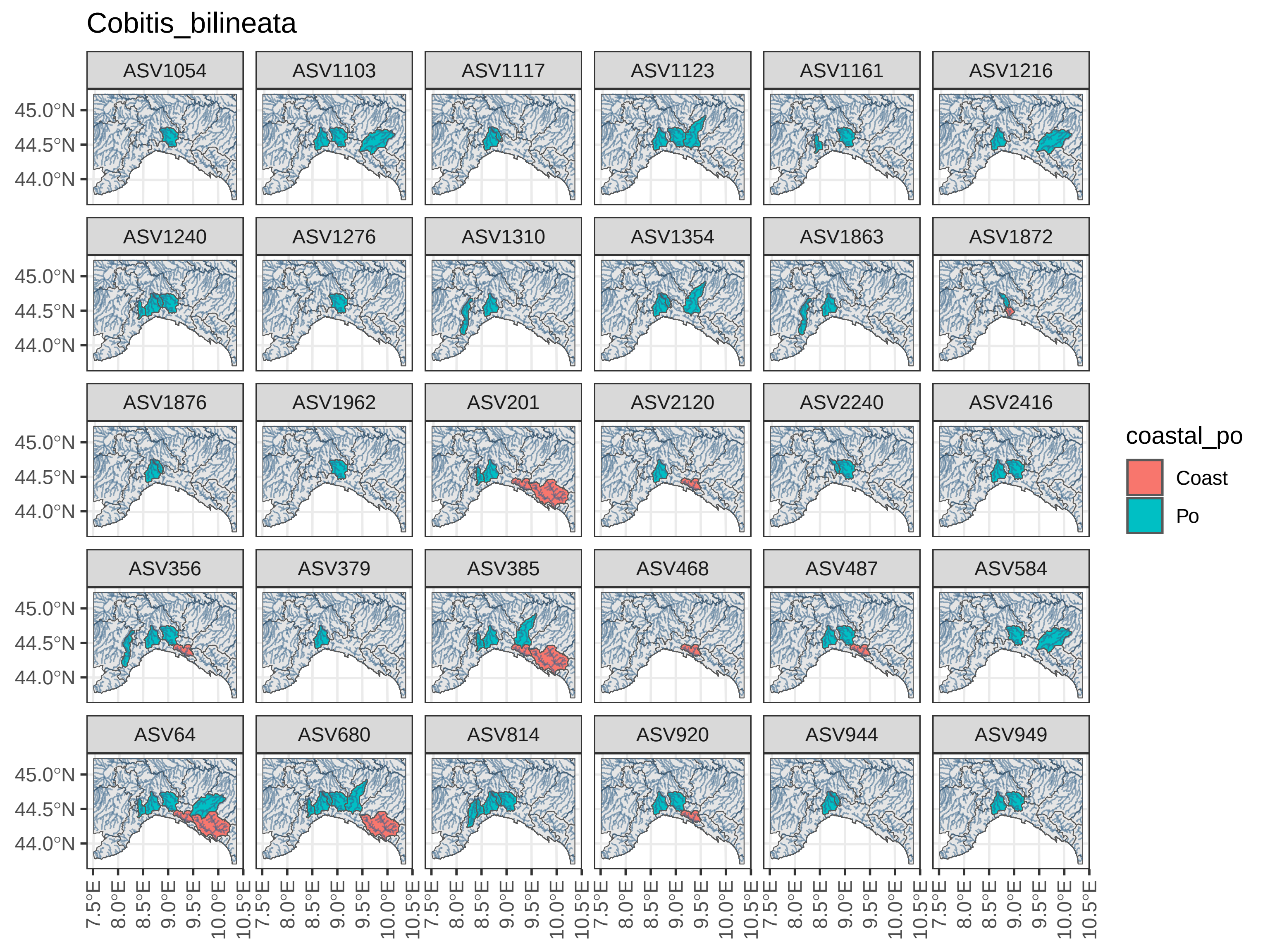
Cobitis_bilineata
ASV1054
ASV1103
ASV1117
ASV1123
ASV1161
ASV1216
ASV1240
ASV1276
ASV1310
ASV1354
ASV1863
ASV1872
ASV1876
ASV1962
ASV201
ASV2120
ASV2240
ASV2416
ASV356
ASV379
ASV385
ASV468
ASV487
ASV584
ASV64
ASV680
ASV814
ASV920
ASV944
ASV949
45.0°N
44.5°N
44.0°N
7.5°E
8.0°E
8.5°E
9.0°E
9.5°E
10.0°E
10.5°E
coastal_po
Coast
Po

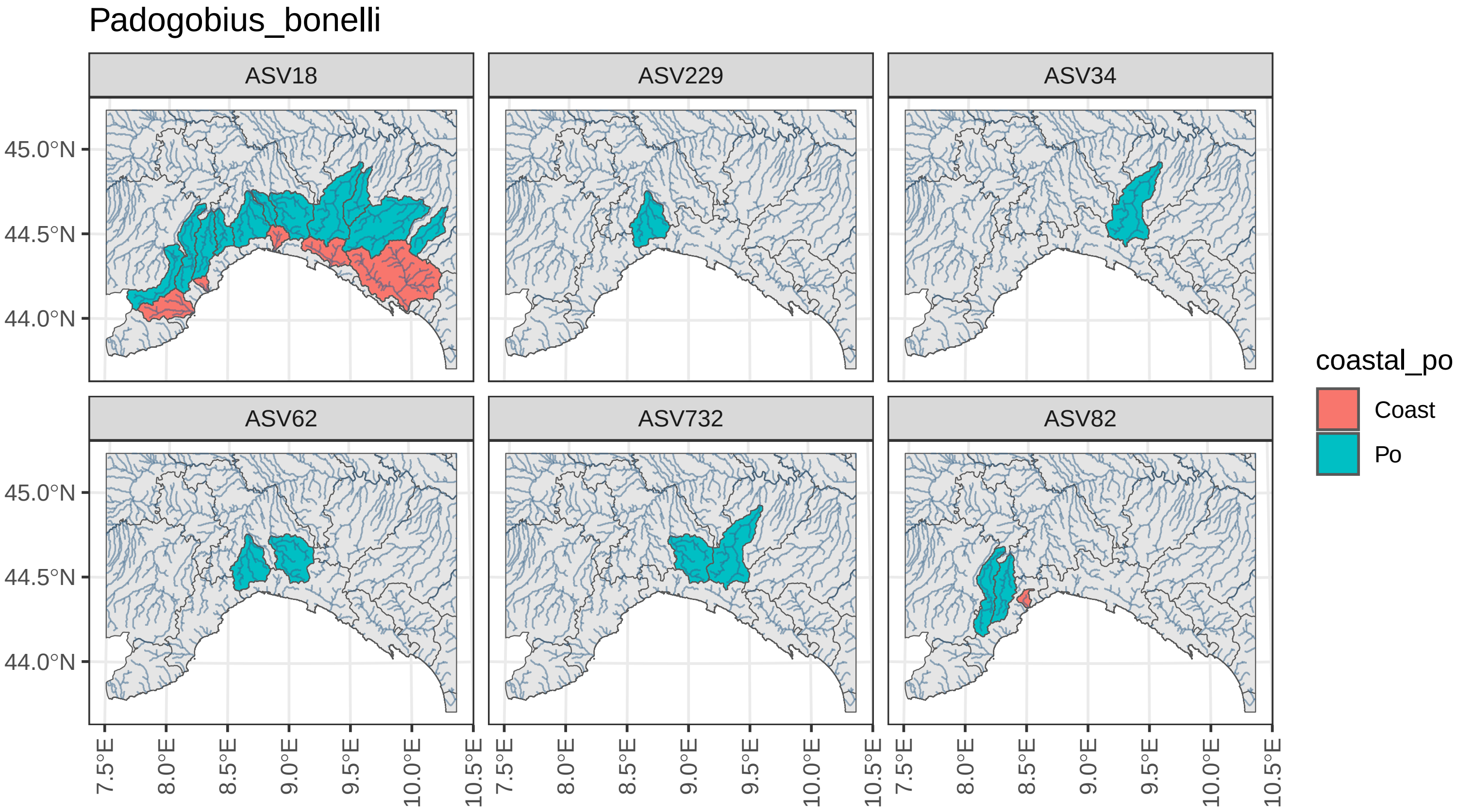

Padogobius_bonelli
ASV18
ASV229
ASV34
ASV62
ASV732
ASV82
45.0°N
44.5°N
44.0°N
7.5°E
8.0°E
8.5°E
9.0°E
9.5°E
10.0°E
10.5°E
coastal_po
Coast
Po

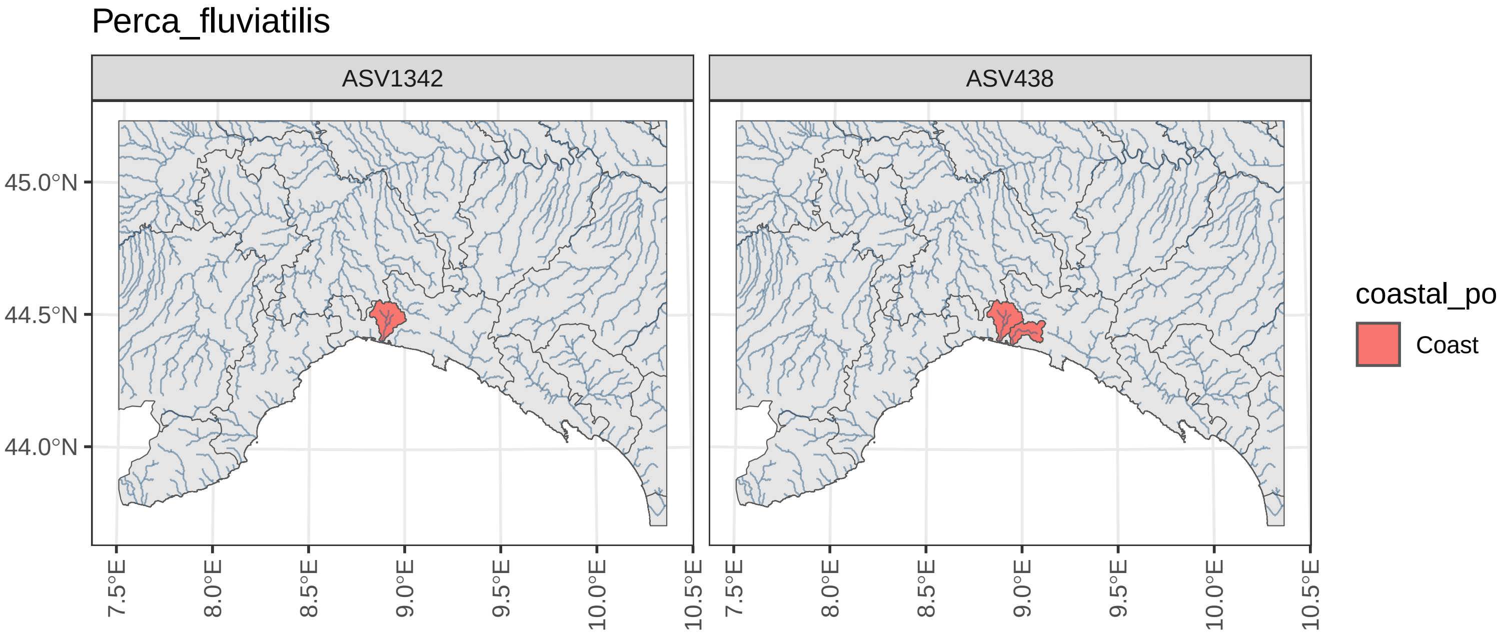
Perca_fluviatilis
ASV1342
ASV438
45.0°N
44.5°N
44.0°N
7.5°E
8.0°E
8.5°E
9.0°E
9.5°E
10.0°E
10.5°E
coastal_po
Coast

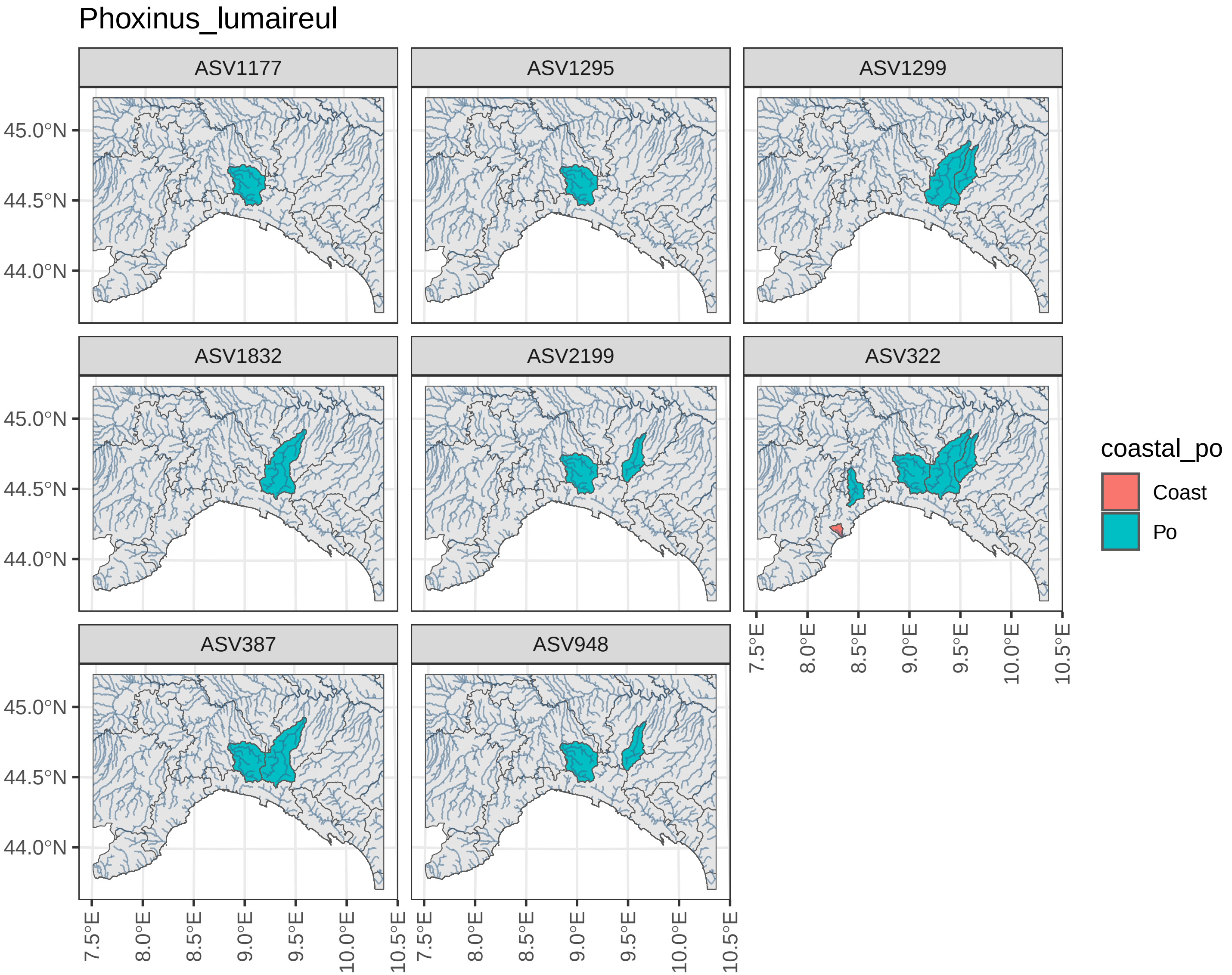

Phoxinus_lumaireul
ASV1177
ASV1295
ASV1299
ASV1832
ASV2199
ASV322
ASV387
ASV948
45.0°N
44.5°N
44.0°N
7.5°E
8.0°E
8.5°E
9.0°E
9.5°E
10.0°E
10.5°E
coastal_po
Coast
Po

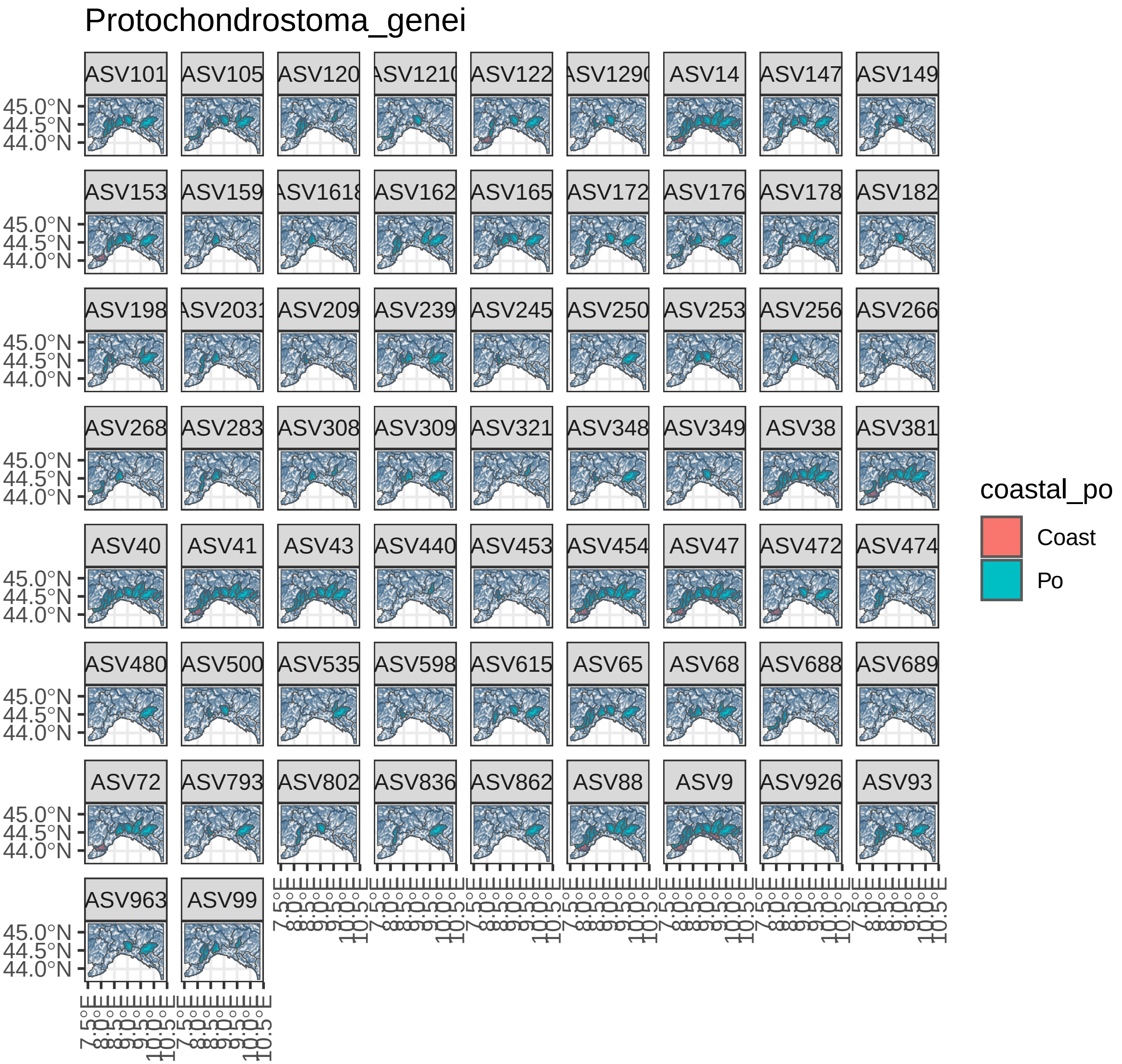

Protochondrostoma_genei
ASV101
ASV105
ASV120
ASV1210
ASV122
ASV1290
ASV14
ASV147
ASV149
ASV153
ASV159
ASV1618
ASV162
ASV165
ASV172
ASV176
ASV178
ASV182
ASV198
ASV2031
ASV209
ASV239
ASV245
ASV250
ASV253
ASV256
ASV266
ASV268
ASV283
ASV308
ASV309
ASV321
ASV348
ASV349
ASV38
ASV381
ASV40
ASV41
ASV43
ASV440
ASV453
ASV454
ASV47
ASV472
ASV474
ASV480
ASV500
ASV535
ASV598
ASV615
ASV65
ASV68
ASV688
ASV689
ASV72
ASV793
ASV802
ASV836
ASV862
ASV88
ASV9
ASV926
ASV93
ASV963
ASV99
45.0°N
44.5°N
44.0°N
coastal_po
Coast
Po

Pseudorasbora_interrupta

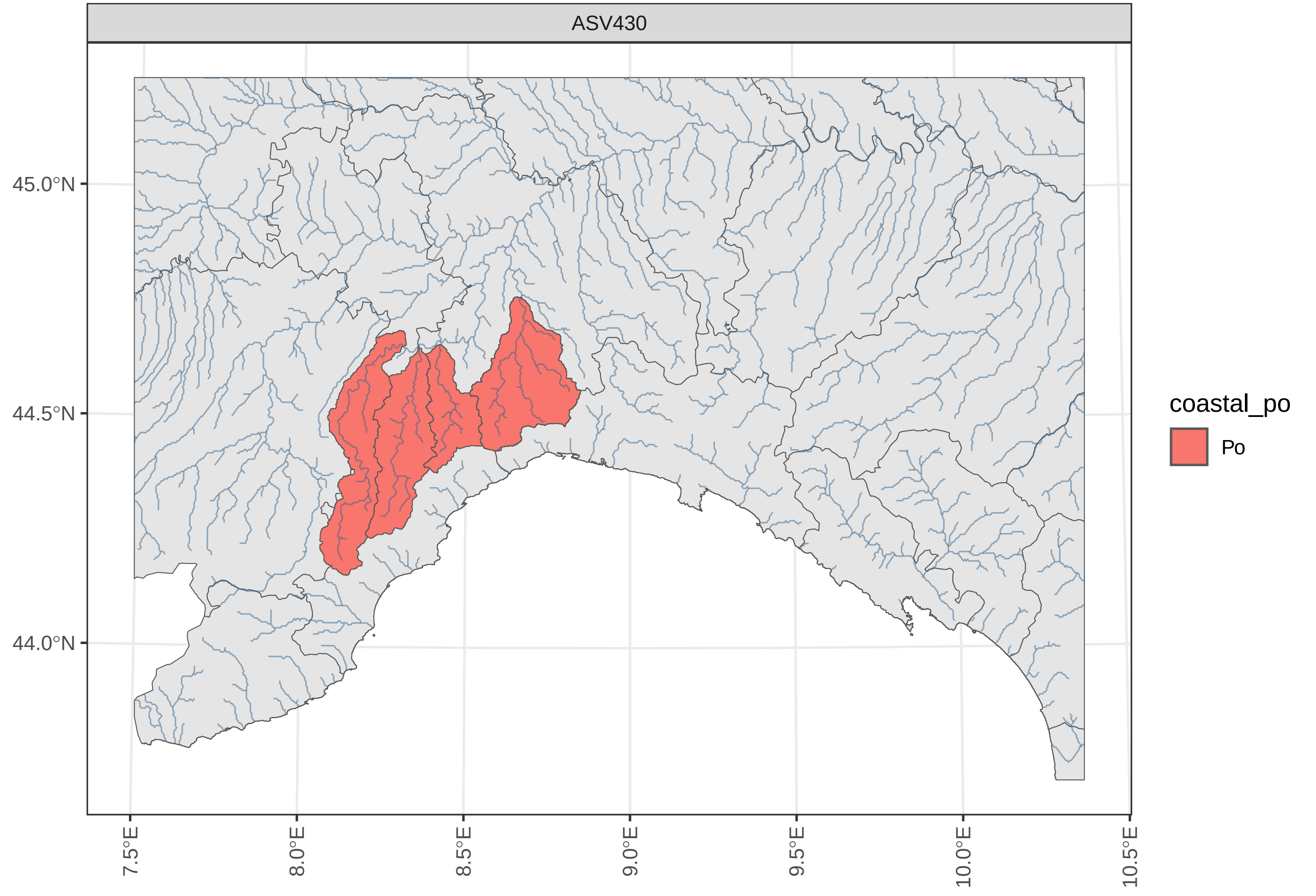

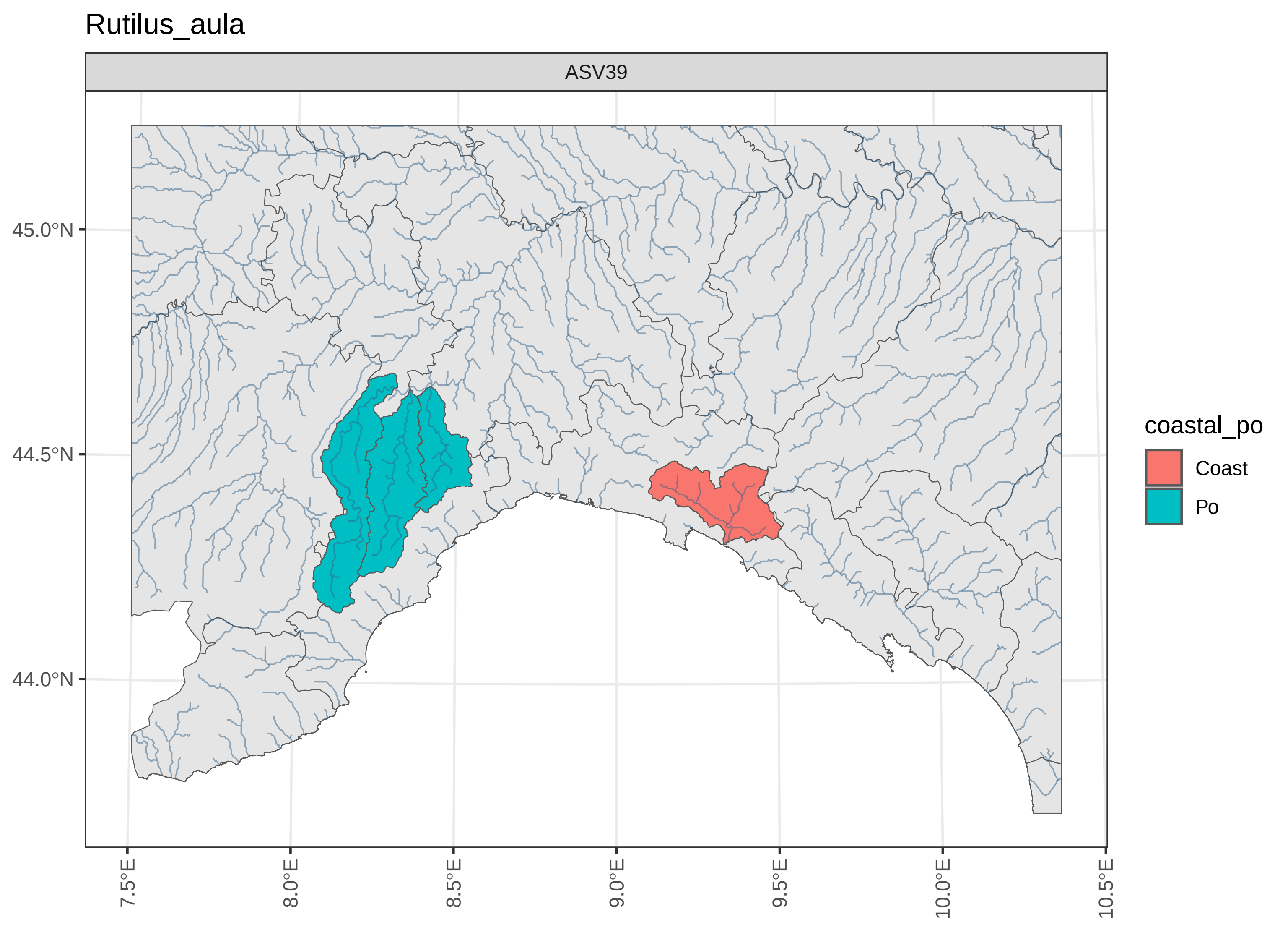
Rutilus_aula
ASV39
45.0°N
44.5°N
44.0°N
7.5°E
8.0°E
8.5°E
9.0°E
9.5°E
10.0°E
10.5°E
coastal_po
Coast
Po

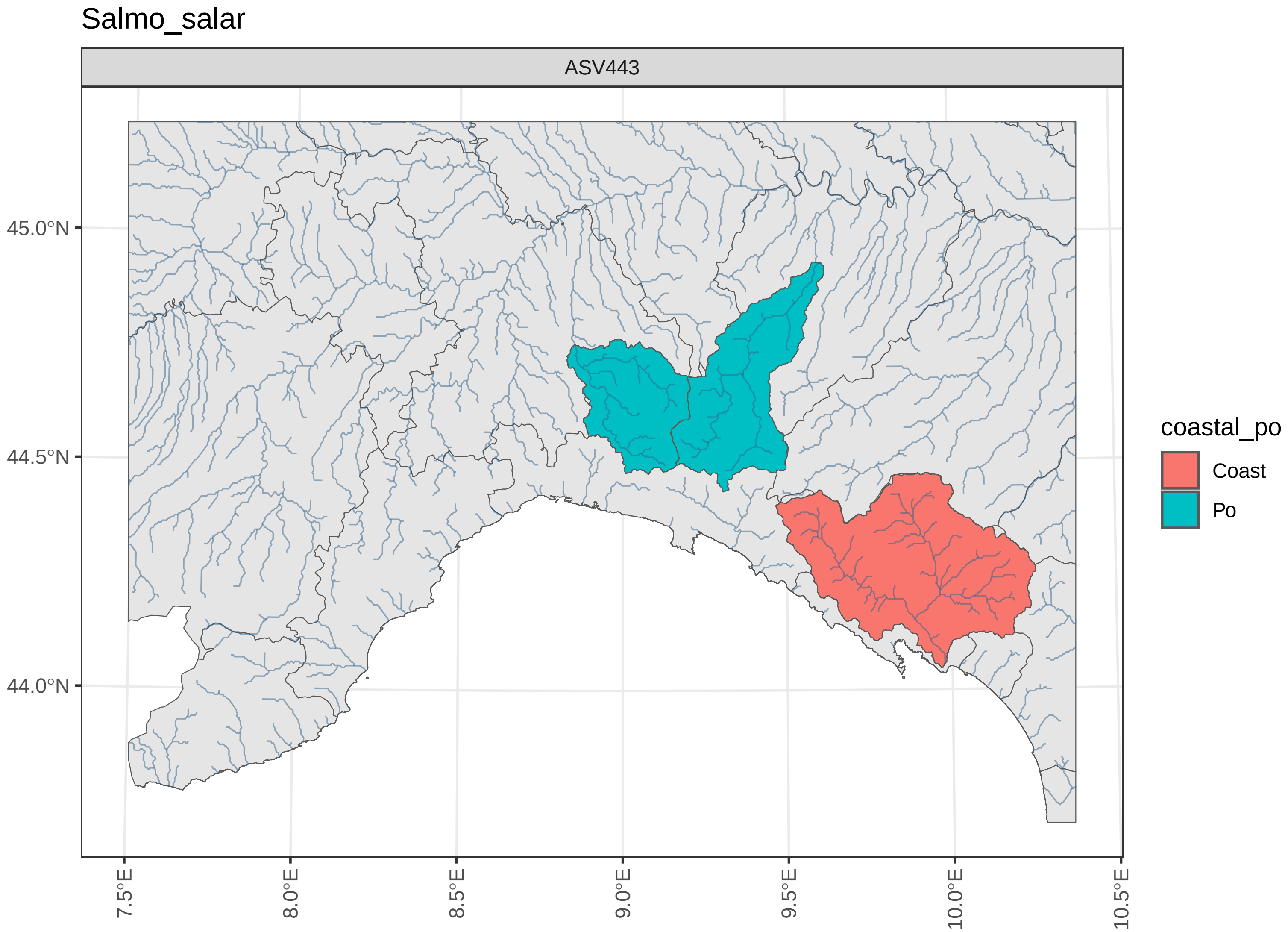

Salmo_salar
ASV443
45.0°N
44.5°N
44.0°N
7.5°E
8.0°E
8.5°E
9.0°E
9.5°E
10.0°E
10.5°E
coastal_po
Coast
Po

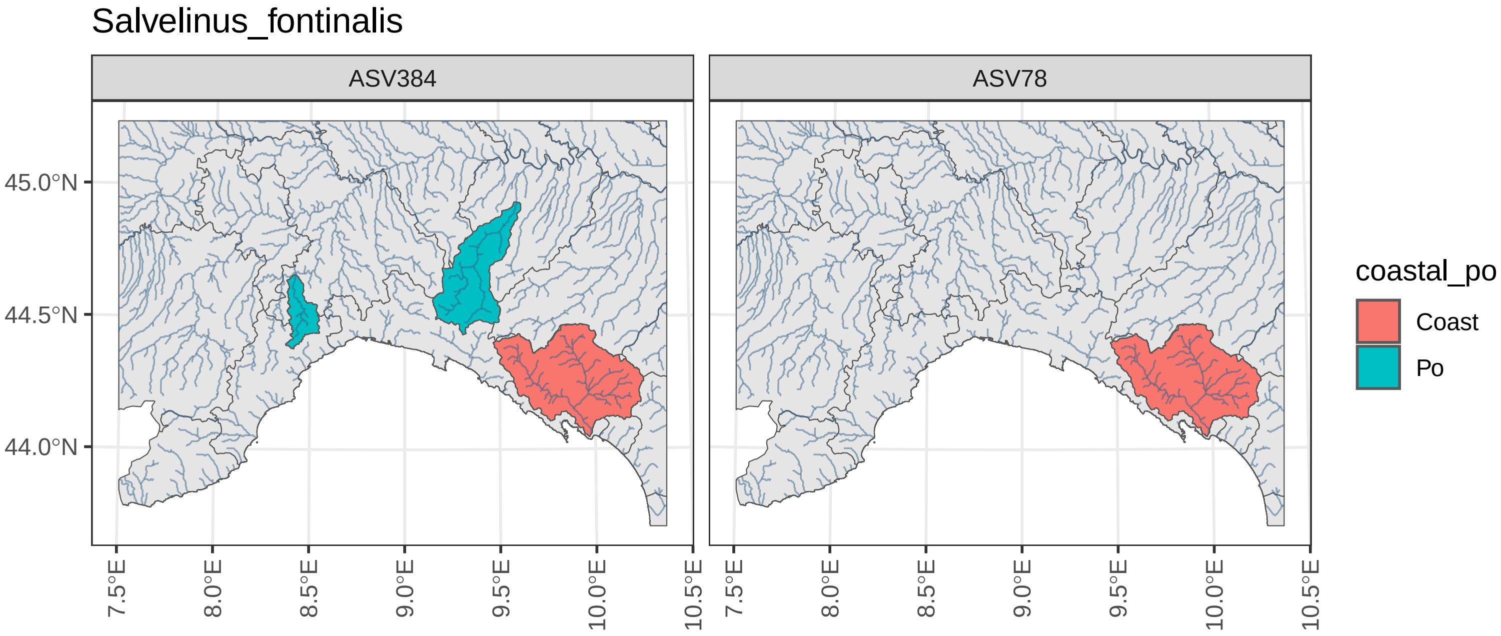

Salvelinus_fontinalis
ASV384
ASV78
45.0°N
44.5°N
44.0°N
7.5°E
8.0°E
8.5°E
9.0°E
9.5°E
10.0°E
10.5°E
coastal_po
Coast
Po

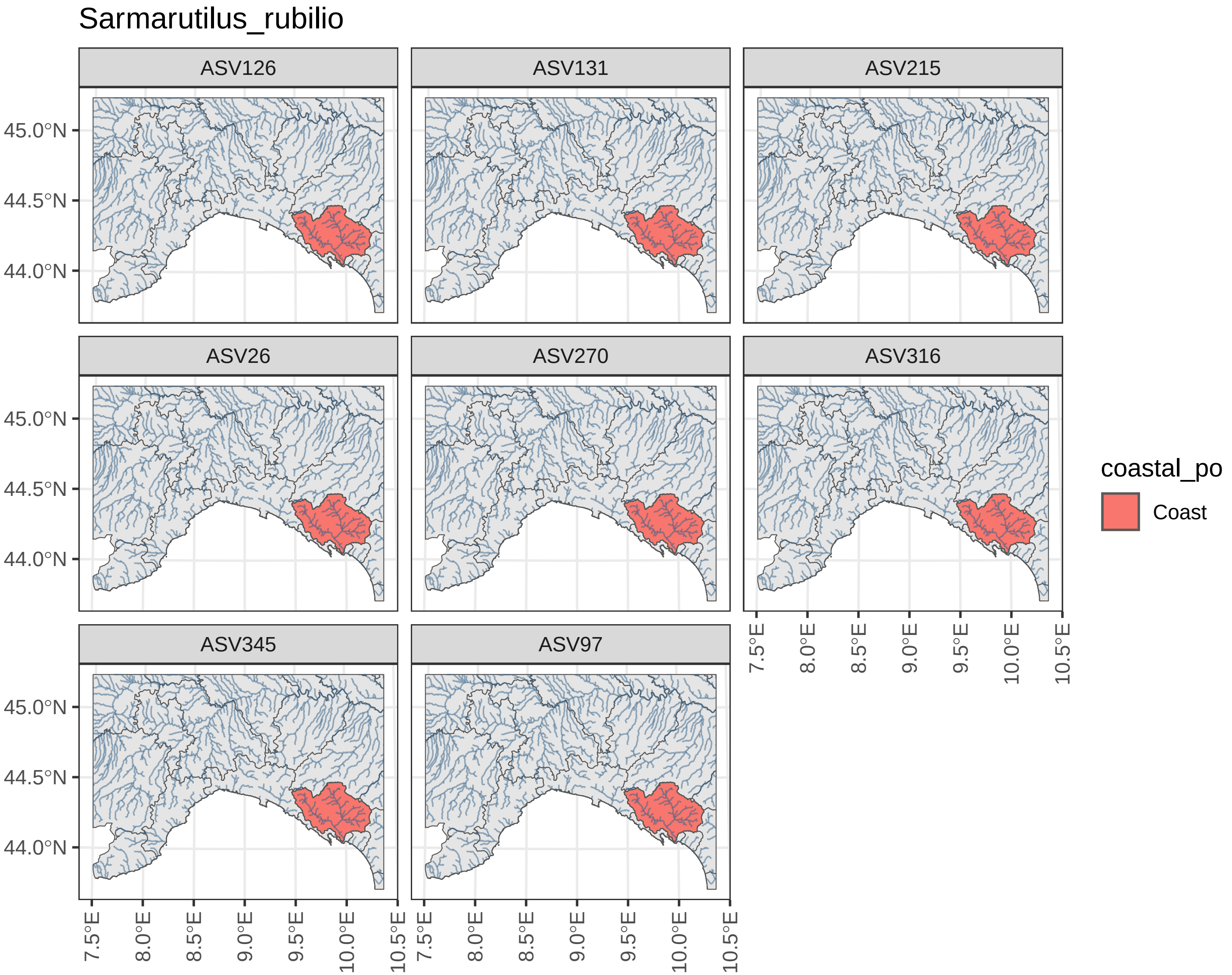

Sarmarutilus_rubilio
ASV126
ASV131
ASV215
ASV26
ASV270
ASV316
ASV345
ASV97
45.0°N
44.5°N
44.0°N
7.5°E
8.0°E
8.5°E
9.0°E
9.5°E
10.0°E
10.5°E
coastal_po
Coast

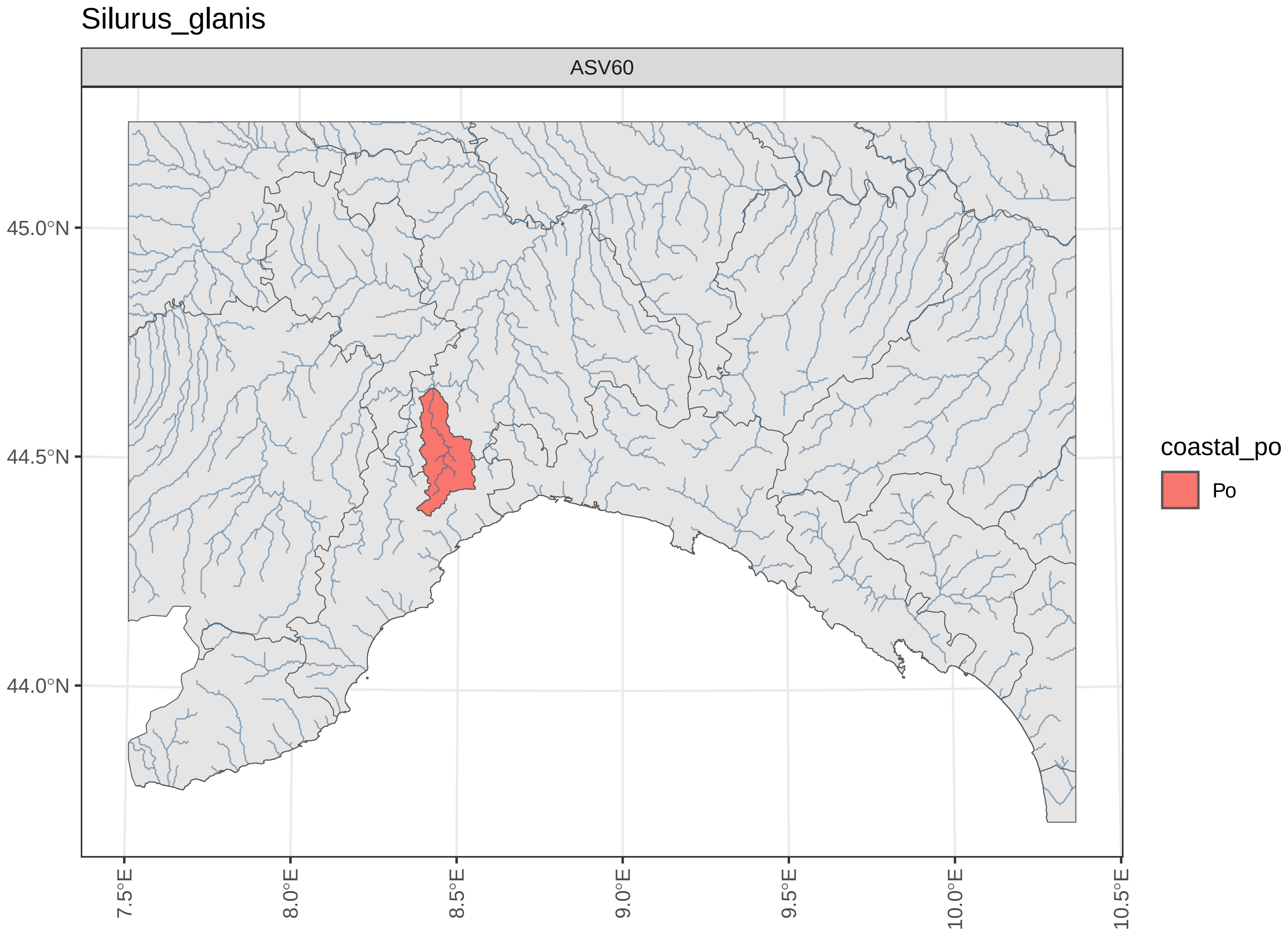

Silurus_glanis
ASV60
45.0°N
44.5°N
44.0°N
7.5°E
8.0°E
8.5°E
9.0°E
9.5°E
10.0°E
10.5°E
coastal_po
Po

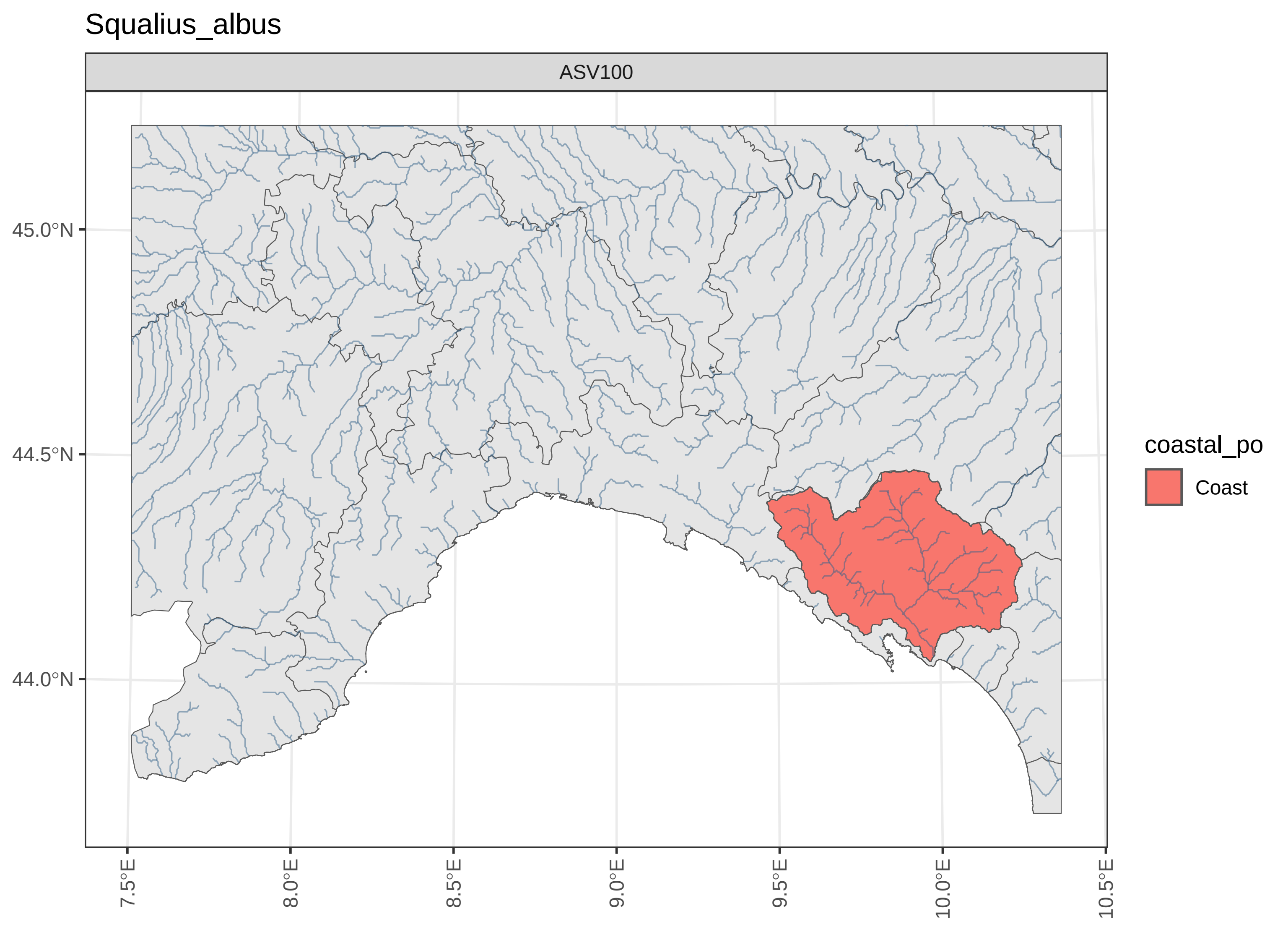

Squalius_albus
ASV100
45.0°N
44.5°N
44.0°N
7.5°E
8.0°E
8.5°E
9.0°E
9.5°E
10.0°E
10.5°E
coastal_po
Coast

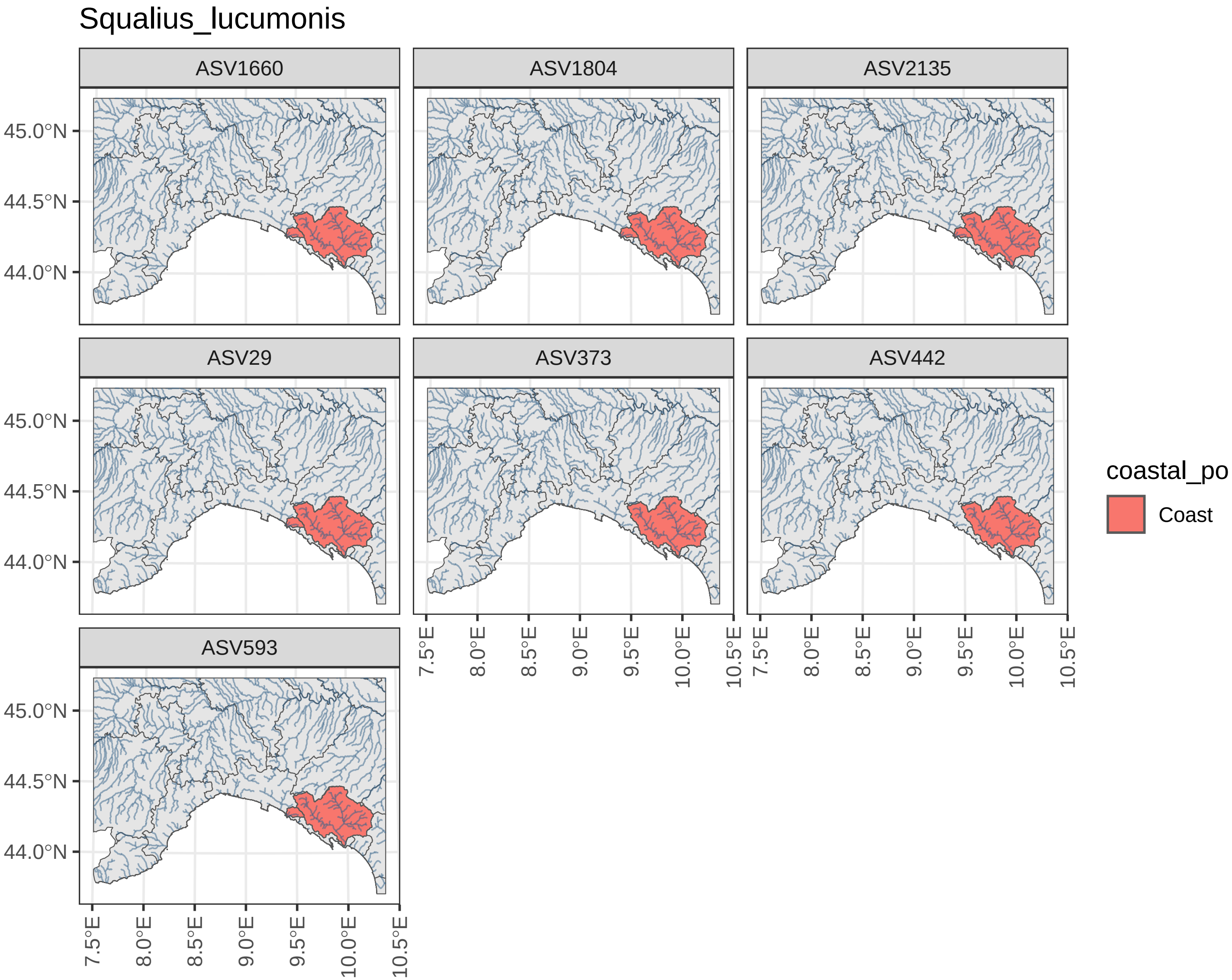
Squalius_lucumonis
ASV1660
ASV1804
ASV2135
ASV29
ASV373
ASV442
ASV593
45.0°N
44.5°N
44.0°N
7.5°E
8.0°E
8.5°E
9.0°E
9.5°E
10.0°E
10.5°E
coastal_po
Coast

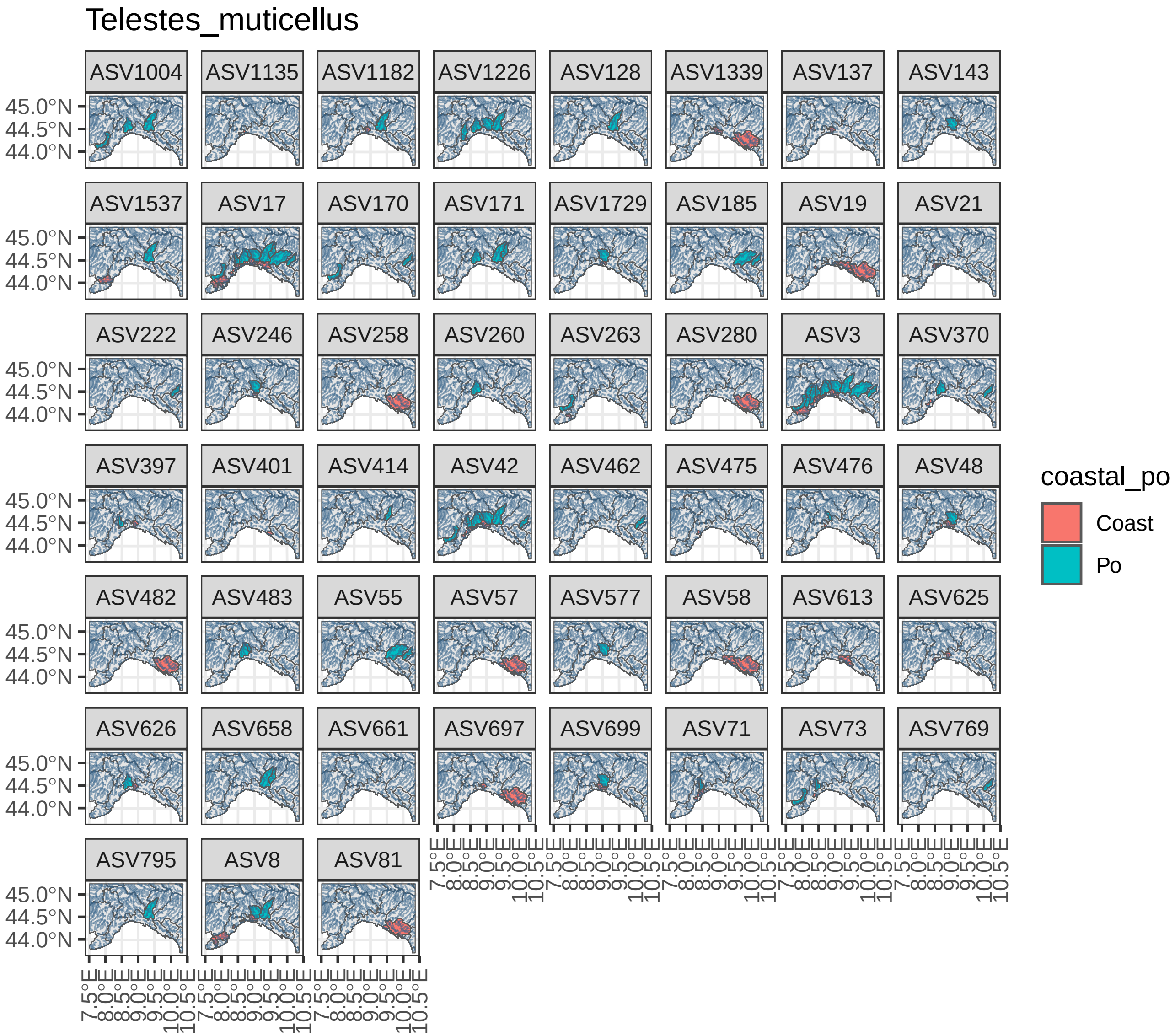
Telestes_muticellus
ASV1004
ASV1135
ASV1182
ASV1226
ASV128
ASV1339
ASV137
ASV143
ASV1537
ASV17
ASV170
ASV171
ASV1729
ASV185
ASV19
ASV21
ASV222
ASV246
ASV258
ASV260
ASV263
ASV280
ASV3
ASV370
ASV397
ASV401
ASV414
ASV42
ASV462
ASV475
ASV476
ASV48
ASV482
ASV483
ASV55
ASV57
ASV577
ASV58
ASV613
ASV625
ASV626
ASV658
ASV661
ASV697
ASV699
ASV71
ASV73
ASV769
ASV795
ASV8
ASV81
45.0°N
44.5°N
44.0°N
7.5°E
8.0°E
8.5°E
9.0°E
9.5°E
10.0°E
10.5°E
coastal_po
Coast
Po
